# A human ex vivo model of radiation-induced skin injury recapitulates p53-driven profibrotic response to radiotherapy

**DOI:** 10.1172/jci.insight.198692

**Published:** 2026-03-19

**Authors:** Caroline Dodson, Sophie M. Bilik, Gabrielle DiBartolomeo, Hannah Pachalis, Lindsey G. Siegfried, Jordan A.K. Johnson, Seth R. Thaller, Irena Pastar, Marjana Tomic-Canic, Anthony J. Griswold, Rivka C. Stone

**Affiliations:** 1 Dr. Phillip Frost Department of Dermatology and Cutaneous Surgery,; 2 The Dr. John T Macdonald Foundation Department of Human Genetics, and; 3 Dewitt Daughtry Family Department of Surgery, Division of Plastic, Aesthetic, and Reconstructive Surgery, University of Miami Miller School of Medicine, Miami, Florida, USA.

**Keywords:** Dermatology, Genetics, Inflammation, Fibrosis, Radiation therapy, p53

## Abstract

Cutaneous radiation injury is an unintended consequence of radiotherapy for many common cancers and can progress to debilitating radiation-induced skin fibrosis (RISF). Existing radiation injury models do not fully capture the skin toxicities observed in patients, contributing to the lack of efficacious therapies to mitigate RISF. To address this, we developed an ex vivo human skin model that recapitulates the temporal radiation injury and RISF response. Human skin explants (*n* = 12) subjected to ionizing radiation demonstrated DNA double-stranded breaks and robust p53-driven transcriptional programming of cell cycle arrest, apoptosis, and senescence compared with nonirradiated controls. Irradiated skin also exhibited induction of pro-inflammatory cytokines, epithelial-mesenchymal transition, profibrotic TGF-β1–mediated signaling, and thickened collagen over time. P53 regulators murine double minute 2 (MDM2) and miR-34a were induced after irradiation and may be leveraged to modulate injury response. Notably, RNA-sequencing of postradiotherapy breast skin from patients who had undergone mastectomy showed similar p53, inflammatory, and TGF-β1 signatures as the ex vivo model, supporting its translational relevance. Together, this model provides a platform for identifying biomarkers and testing therapies to prevent or mitigate cutaneous radiation toxicities. Targeting the dynamic p53-driven profibrotic radiation response represents a potentially new therapeutic avenue to improve quality of life for patients after radiotherapy.

## Abstract 2

We developed a human skin model that recapitulates radiotherapy-induced fibrosis, uncovering a p53-driven fibrotic program and guiding strategies to mitigate skin radiation injury in cancer survivors.

## Graphical abstract

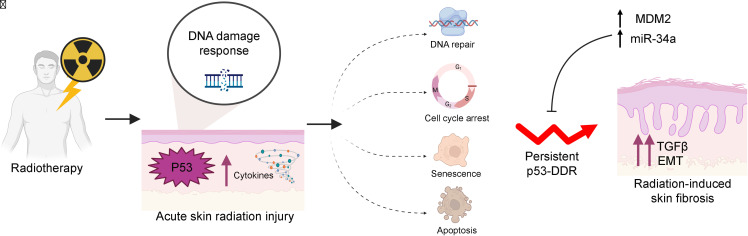

## Introduction

Radiotherapy is a critical component of cancer treatment that confers survival benefit for many common cancers, with at least 50% of oncology patients receiving ionizing radiation as part of their care (1). A substantial consequence of radiotherapy is collateral damage to healthy tissues, particularly the skin, which lies in the path of the ionizing radiation beam as it travels to target and destroy tumor cells (2, 3). Approximately 95% of patients experience various skin reactions to radiotherapy, which range from mild to severe and are influenced by dose, overlapping fields, and treatment duration (4). Although most acute reactions resolve within days to weeks, 30%–70% of patients experience a continuous skin injury response, which progresses to chronic, debilitating, radiation-induced skin fibrosis (RISF) resulting in disability, pain, and diminished quality of life in the months to years following radiation exposure (4–6). Current clinical management of cutaneous radiation toxicities focuses on symptomatic relief of acute skin reactions through supportive measures, such as topical emollients, hyaluronic acid–based compounds, and corticosteroids in high-risk patients, as well as lifestyle changes like avoiding sun exposure and skin irritants (7, 8). However, no therapy has demonstrated efficacy in halting progression to RISF (2). Moreover, although primary or immortalized cells and animal models recapitulate some mechanistic and phenotypic features of RISF (9–11), translation of these findings to clinical therapies has not yet succeeded, underscoring the critical need for more translationally relevant systems. A deeper understanding of pathological processes driving the evolution of RISF in intact human skin tissue is needed to predict, mitigate, and treat skin fibrosis in patients receiving lifesaving radiotherapy.

As a self-renewing proliferative organ, the skin is particularly vulnerable to ionizing radiation, which disproportionately affects rapidly dividing cells (12). Specifically, basal keratinocytes, hair follicle stem cells, and melanocytes are highly radiosensitive, accounting for the alopecia and pigmentary skin changes that are commonly observed after radiotherapy (4, 13, 14). Radiation-induced cellular damage initiates a cascade of events resembling phases of the classic wound healing response to barrier compromise (i.e., acute wounds), including inflammation, keratinocyte proliferation, and remodeling aimed at tissue repair (14–16).

One of the earliest and most critical events in the radiation-specific cascade is the DNA damage response (DDR), which is triggered by both direct and indirect mechanisms (17). Ionizing radiation directly causes DNA double-stranded breaks, leading to the activation of the ataxia telangiectasia mutated (ATM) kinase and subsequent phosphorylation of histone H2AX (18–21). Indirectly, radiation generates free radicals that contribute to additional DNA damage and oxidative stress (12, 22). Among the key downstream effectors of ATM is the transcription factor p53, which regulates cell fate decisions after radiation injury — promoting cell cycle arrest to allow DNA repair or initiating senescence or apoptosis when repair is unsuccessful (19, 23–26). Fibrosis evolves after radiation as it does in many other organs and tissues: unrepaired injury drives chronic inflammation that perpetuates TGF-β activation, collagen production, and excess extracellular matrix (ECM) deposition in the skin, leading to RISF (2–4, 7, 27). To better understand these complex processes, we present a human ex vivo model of radiation-induced injury designed to emulate the cutaneous response to ionizing radiation observed in patients after radiotherapy, spanning the continuum from acute injury and inflammation to progressive fibrosis. We focused on recapitulating 4 elements underpinning this complex response: (a) the DDR, (b) the role of p53 and its regulators such as murine double minute 2 (MDM2) and miR-34a in modulating the radiation injury response, (c) the pro-inflammatory cytokine cascade, and (d) profibrotic signaling and fibrotic phenotype. By highlighting the model’s clinical implications through a comparative review with irradiated skin profiles from patients with breast cancer, we posit this model as a reliable and translatable tool for investigating the primary early drivers of RISF and for evaluating potentially new therapeutic modalities.

## Results

### Induction of DNA damage by ionizing radiation in human skin ex vivo.

**Figure 1:**
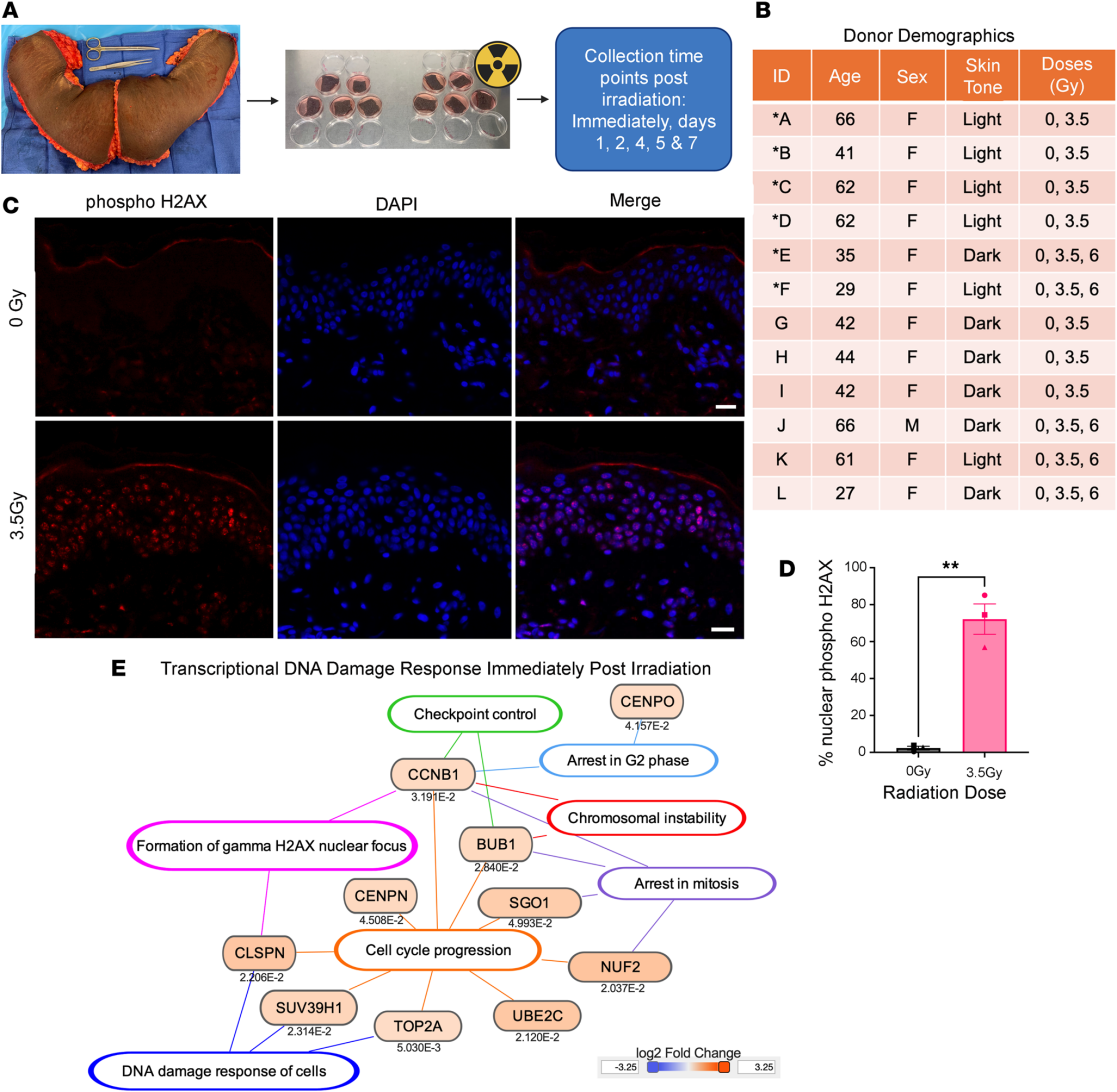
Establishment of a human ex vivo skin model and validation of early DNA damage responses to study radiation-induced injury. (**A**) Workflow schematic of the human ex vivo model used in this study. (**B**) Donor demographics (ID, age, and sex) and irradiation dose. Donors A–F, marked with an asterisk, were used for bulk RNA-seq. (**C**) Representative immunofluorescence images at 1 hour after irradiation showing phosphorylated H2AX (red) as a marker of dsDNA breaks, DAPI (blue) nuclear counterstain, and merged images from irradiated (3.5 Gy) and control (nonirradiated) samples. Scale bar: 20 μm. (**D**) Quantification of H2AX foci staining from **C**, based on counts from 3 independent blinded observers. Data represents *n* = 3 donors; symbols correspond to individual donors; mean ± SD. Statistical significance was assessed using a 1-tailed paired *t* test. (**E**) IPA of gene expression data in skin immediately after irradiation, highlighting cell survival–related genes and pathways. Differential expression was performed using edgeR on bulk RNA-seq data filtered for protein-coding genes. IPA Core Analysis was filtered to genes with *P* ≤ 0.05 and log_2_ fold-change ≥ ±0.5. Gene color reflects *z* score; numerical values indicate Benjamini-Hochberg–corrected –log_10_ (*P* value). Asterisks indicate statistical significance (*P < 0.05, **P < 0.01).

To investigate the molecular mechanisms driving the cutaneous radiation injury response, we developed a human ex vivo model in which skin obtained from patients undergoing elective panniculectomy procedures was irradiated with a single dose of 0 (nonirradiated), 3.5, or 6 Gy and maintained in culture at an air-liquid interface for 7 days. Nonirradiated skin from each donor was maintained under identical conditions and served as a control, minimizing interdonor variability and ensuring observed changes were not attributable to being in culture alone (Figure 1A). Irradiated and control skin from a total of 12 donors (Figure 1B) was collected immediately (30–60 min) and on days 1, 2, 4, 5, and 7 after irradiation. We then profiled the cellular and molecular responses to radiation injury with a focus on epidermal keratinocytes, dermal fibroblasts, and skin-resident immune cells.

As initial validation of our ex vivo model, we confirmed the induction of DNA double-stranded breaks after irradiation by using immunofluorescence (IF) staining for phosphorylated H2AX, an established marker of DNA double-stranded breaks (18, 19). Irradiated skin displayed positive nuclear staining of H2AX foci compared with controls immediately after irradiation (Figure 1, C and D). This was largely resolved by 24 hours (1 day) after irradiation, reflecting efficient repair of catastrophic DNA damage (28).

Having established the anticipated induction and repair of DNA damage following ionizing radiation, we proceeded to perform bulk RNA-seq on skin from 6 donors to characterize the broader, time-dependent radiation-induced response in our model. Transcriptomic profiles from irradiated (3.5 Gy) and control skin were compared at each time point: immediate (30–60 min) and days 1, 2, 5, and 7 after irradiation. Differentially expressed genes meeting significance thresholds of nominal *P* value of 0.05 or less and log_2_ fold-change of ±0.5 or greater (Supplemental Figure 1; supplemental material available online with this article; https://doi.org/10.1172/jci.insight.198692DS1) were subjected to IPA (QIAGEN). Importantly, the early transcriptional signature immediately after irradiation reflected the canonical acute DDR (28), evidenced by significant upregulation of genes and pathways related to DNA damage, chromosomal instability, cell cycle arrest, and formation of H2AX foci (Figure 1E), consistent with our tissue staining in Figure 1C. This initial validation substantiates that our ex vivo model replicates the initial radiation-induced molecular events as expected, thereby providing a robust platform for comprehensive mechanistic investigations into the progression of acute to chronic radiation injury.

### P53 drives the DDR to ionizing radiation in human ex vivo skin.

**Figure 2:**
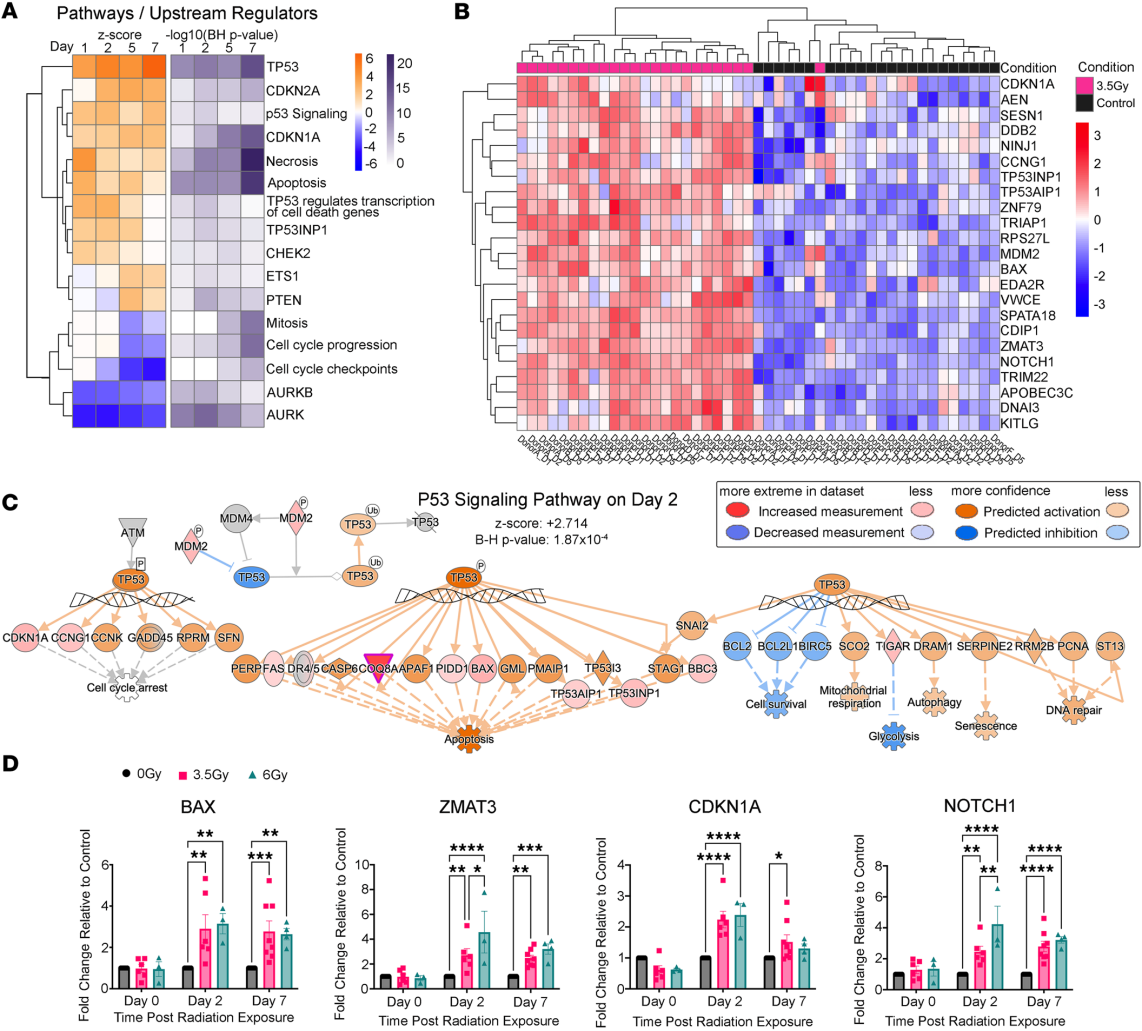
p53-mediated DNA damage response in irradiated ex vivo skin. (**A**) IPA of predicted upstream regulators and biological pathways associated with DNA damage responses across days 1, 2, 5, and 7 after irradiation. The first heatmap is colored by IPA *z* score (predicted activation state); the second shows Benjamini-Hochberg–corrected –log_10_ *P* values (e.g., 1.3 corresponds to *P* ≤ 0.05). Differentially expressed genes were filtered for protein-coding, *P* ≤ 0.05, and log_2_ fold-change ≥ ±0.5. *z* score heatmap rows were clustered by decreasing row average, with *P* value heatmap rows ordered to match. (**B**) Heatmap of *TP53*-regulated genes from RNA-seq data, showing row-scaled log_2_-transformed counts per million (CPM) values. Red indicates increased expression; blue indicates decreased expression. Columns represent individual samples labeled by donor and time point (e.g., DonorA_D1, DonorB_D7). Annotation bars indicate condition above the heatmap (pink = 3.5 Gy, black = control). (**C**) IPA-annotated “p53 signaling pathway” overlaid with RNA-seq gene expression changes on day 2 after irradiation. Genes are colored by predicted activation state (orange = activated, blue = inhibited). (**D**) qPCR of selected p53 target genes in human ex vivo skin on days 0, 2, and 7 after radiation of 0 Gy (black, *n* = 6), 3.5 Gy (pink, *n* = 6), or 6 Gy (teal, *n* = 3–4). Data were analyzed using the ΔΔCt method, normalized to *GAPDH*, and presented as fold-change relative to matched 0 Gy controls for each donor and time point; mean ± SD. Statistical significance was assessed using a mixed-effects model with Tukey’s test for multiple comparisons. Asterisks indicate statistical significance (*P < 0.05, **P < 0.01, ***P < 0.001, ****P < 0.0001). All heatmaps were generated in RStudio using the pheatmap package.

To further explore the radiation injury response in our ex vivo model over time, we utilized IPA’s Comparison Analysis tool, in which pathway analyses from days 1, 2, 5, and 7 (enrichment *P* values and *z* scores for pathways, processes, and upstream regulators) were compared side by side and grouped by similarity (Figure 2A). The top common upstream regulator induced and activated across all days after irradiation was *TP53*. Other pathways and processes involving p53 signaling and cell death were similarly activated over time, while processes and regulators of cell cycle progression like Aurora kinases that control entry into mitosis (29) showed predicted inhibition. These findings indicate that our ex vivo model captures an early, robust, and sustained p53-mediated DDR to ionizing radiation. This is consistent with previous studies demonstrating the central role of p53 in driving the radiation response in both healthy and tumor tissues (23–25, 30, 31).

We then visualized the expression patterns of *TP53*-regulated genes over time (Figure 2B), revealing distinct clusters of upregulated genes whose induction was consistent across transcriptomic profiles of all 6 human donors. To visualize the functional context for these genes, we overlaid the postirradiation day 2 profiles on IPA’s canonical p53 signaling pathway with associated enrichment *P* value of 1.87 × 10^–4^ and activation *z* score of +2.714 (Figure 2C). In this annotated pathway, the directionality (upregulation or downregulation) of the differentially expressed genes predicted activation of apoptosis, autophagy, senescence, and DNA repair processes, while cell survival and glycolysis/metabolism were predicted to be inhibited after irradiation. Taken together, pathway analyses indicated that p53 is the core driver of the DDR to radiation in our ex vivo model, thereby recapitulating findings from in vivo tissues (24, 25, 32–35). This further validates our human ex vivo model as a reliable platform for studying cutaneous radiation injury but also supports its utility for characterizing key cellular fates and testing interventions that modulate the p53 pathway.

To validate the RNA-seq findings, we selected a subset of p53 target genes with central roles in the canonical DDR: BCL2 associated X (*BAX*) and zinc finger matrin-type 3 (*ZMAT3*) in apoptosis, cyclin dependent kinase inhibitor 1A (*CDKN1A*; p21) in cell cycle arrest, and notch receptor 1 (*NOTCH1*) in p53 suppression (24, 36, 37). We explored the temporal and dose-dependent expressions of these genes in an expanded pool of 8 donors treated with either 3.5 Gy or 6 Gy radiation. qPCR findings confirmed significant upregulation of these genes on day 2 and day 7 after irradiation (Figure 2D and Supplemental Figure 2). We further observed that the 6 Gy dose elicited a more pronounced response compared with 3.5 Gy, and *ZMAT3* and *NOTCH1* displayed a significant dose-dependent response 2 days after irradiation. These findings further support the evolving temporal activation of p53 signaling and the induction of its downstream targets in our ex vivo human skin model.

### Autoregulation of the p53-mediated DDR by MDM2 and miR-34a-5p.

**Figure 3:**
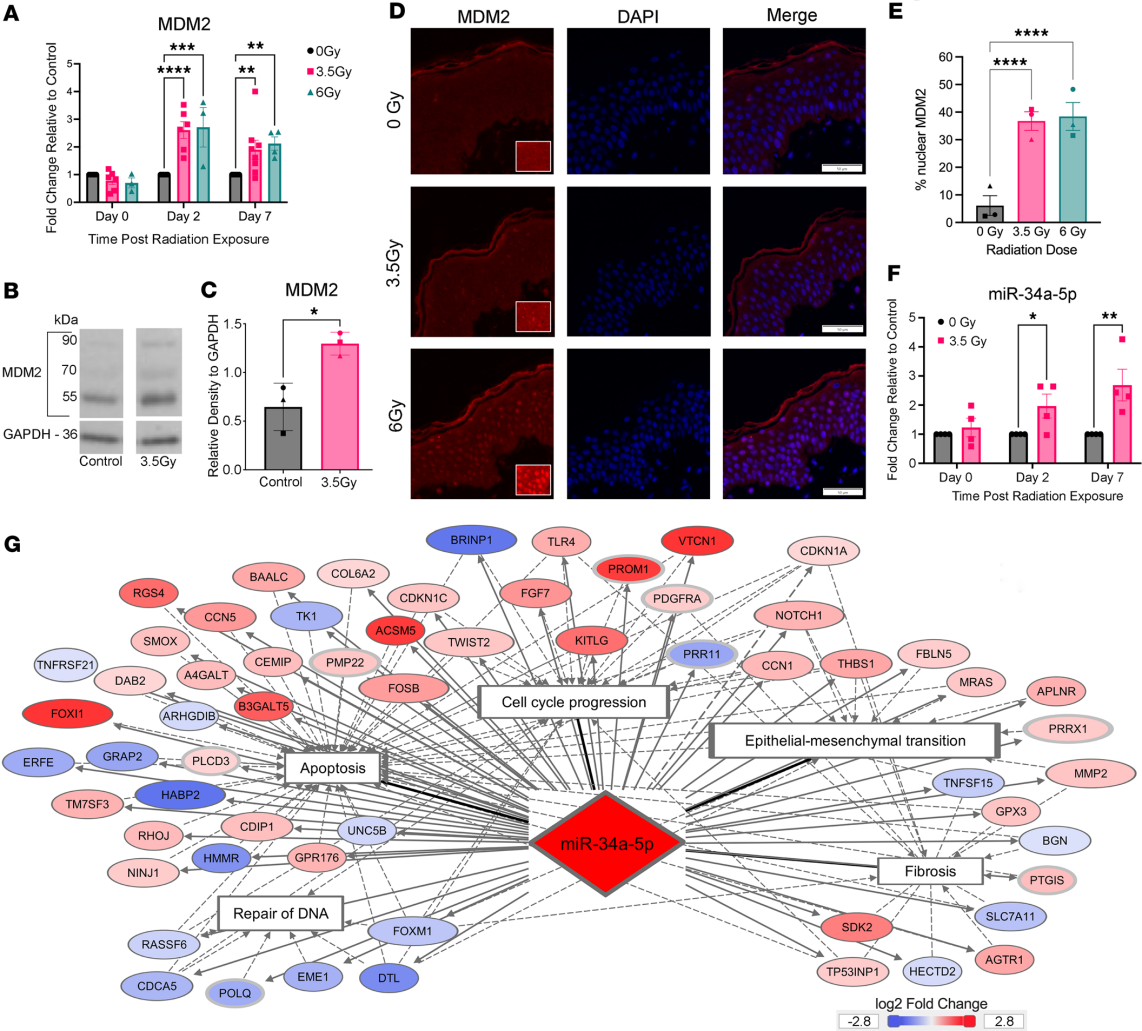
Regulators of p53-mediated DNA damage response. (**A**) qPCR of *MDM2* expression in human ex vivo skin with 0 Gy (black, *n* = 6), 3.5 Gy (pink, *n* = 6), and 6 Gy (teal, *n* = 3–4) on days 0, 2, and 7 after irradiation. Expression was normalized to *GAPDH*. (**B**) Representative Western blot showing MDM2 protein expression on day 7 in control and 3.5 Gy samples. GAPDH served as the loading control. Lanes were run on the same gel but were noncontiguous. (**C**) Quantification of MDM2 protein levels in **B**. Band intensities were extracted using ImageJ (NIH); multiple MDM2 isoforms were averaged for each donor (*n* = 3); normalized to GAPDH. (**D**) Representative immunofluorescence staining of phospho-MDM2 (Ser395) on day 1 after irradiation, showing cytoplasmic localization in control and increased nuclear localization in irradiated samples (3.5 and 6 Gy). Scale bar: 50 μm. (**E**) Quantification of phospho-MDM2 nuclear localization from **D**, expressed as the percentage of cells with nuclear localization. Data were collected by 3 independent blinded observers and analyzed using 2-way ANOVA with Tukey’s multiple-comparison test (*n* = 3; symbols correspond to individual donors). (**F**) qPCR analysis of mature miR-34a-5p expression using small RNA-enriched cDNA. Expression was normalized to SNORD48. Data representative of *n* = 6. (**G**) IPA generated network of miR-34–regulated genes on day 7 after irradiation. Functional annotations highlight associations with apoptosis, cell cycle, fibrosis, EMT, and DNA repair. Asterisks indicate statistical significance (*P < 0.05, **P < 0.01, ***P < 0.001, ****P < 0.0001). For **A** and **F**, after normalization, data were analyzed using the ΔΔCt method and presented as fold-change relative to the 0 Gy control at each time point, and statistical significance was assessed using a mixed-effects model with Tukey’s test for multiple comparisons; mean ± SD.

We then shifted to explore regulators of the p53-mediated DDR in our model, which might serve as therapeutic targets to modulate the radiation injury response (38). *MDM2* is a well-established negative regulator of p53 signaling that undergoes posttranslational modification followed by nuclear translocation during the p53-mediated DDR (25, 39–41). Bulk RNA-seq revealed differential expression of *MDM2* at 3.5 Gy, which was further supported by qPCR showing an early and dose-dependent induction of *MDM2* on day 2 across 3 radiation doses (0, 3.5, and 6 Gy; Figure 3A). Western blot analysis identified an increase in MDM2 isoforms (55–90 kDa) on day 7 after irradiation (Figure 3, B and C). DNA damage–induced phosphorylation of *MDM2* at Ser395 by ATM kinase triggers its nuclear localization, where it binds and inhibits transactivation of p53 (42). IF staining using an anti-phospho serine 395-MDM2 antibody revealed MDM2 nuclear accumulation in irradiated tissue compared with nonirradiated controls (Figure 3, D and E).

We also noted that *MIR34AHG*, the gene encoding miR-34a, was significantly upregulated in our RNA-seq dataset across donors and over time (Supplemental Figure 3). MiR34a is a p53-induced noncoding RNA that sensitizes cells to p53-mediated apoptosis following radiation damage, thereby enhancing tissue radiosensitivity (25, 43, 44). It has also been identified as a potential target to protect tissue from radiation damage (25, 44, 45). Using an miR-specific qPCR assay, we validated the induction of miR-34a-5p after irradiation, which was most significant on day 7 (Figure 3F). To explore the functional consequences of miR-34a induction in our model, we used IPA miRNA Target Filter analysis to identify miR-34a targets among the genes regulated on day 7 after irradiation in our RNA-seq dataset and to determine the biological processes associated with these targets (Figure 3G). The resulting network encompassed DNA damage repair, apoptosis, and cell cycle regulation as anticipated, but also highlighted miR-34a’s role in the fibrotic response (46). Specifically, miR-34a target genes involved in epithelial-mesenchymal transition (EMT) and fibrosis were upregulated or downregulated 7 days after irradiation (Figure 3G), linking the early (p53-mediated DDR) and late (profibrotic) radiation response in our ex vivo model.

### Radiation induces pro-inflammatory cytokines in ex vivo skin.

**Figure 4:**
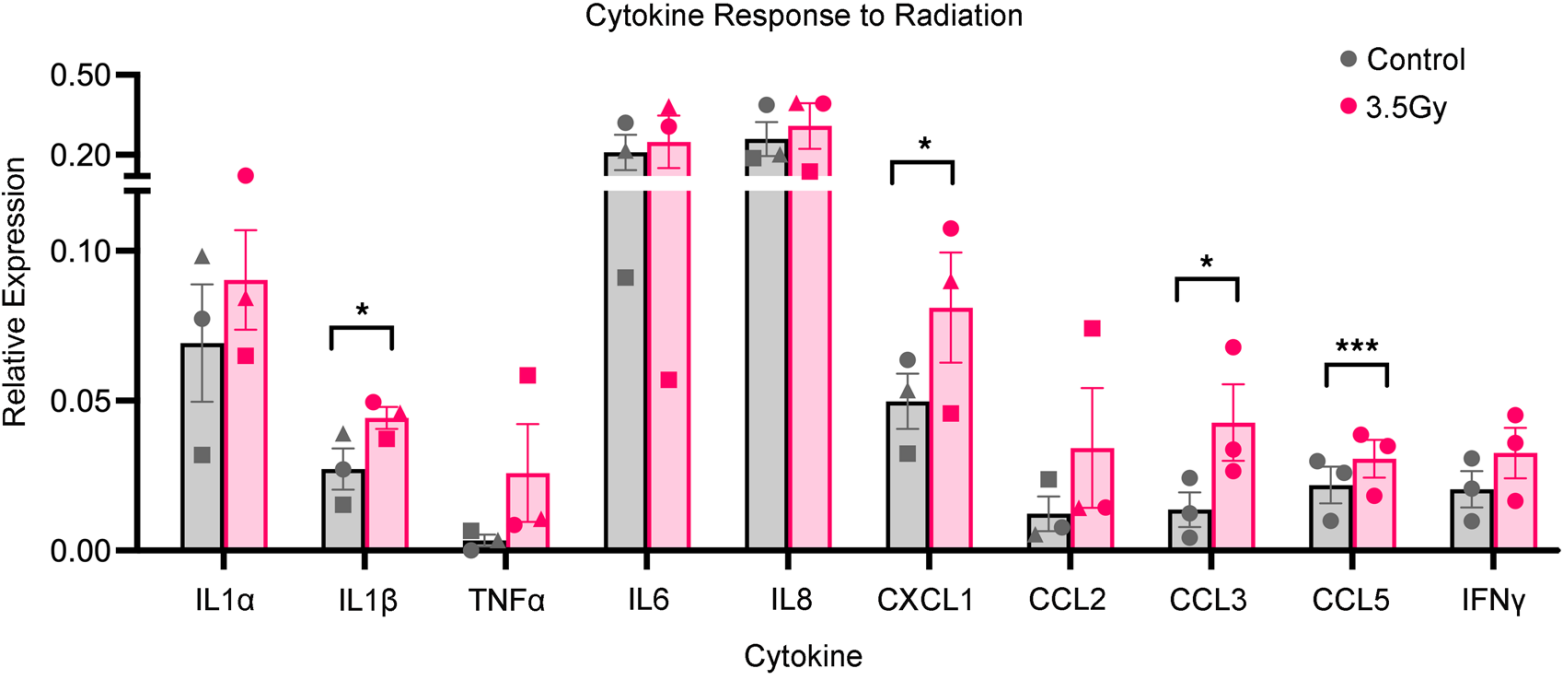
Upregulation of pro-inflammatory cytokines in ex vivo irradiated human skin. Cytokine array quantification of 3.5 Gy irradiated and control skin on day 7 after irradiation. Asterisks indicate statistical significance (*P < 0.05, ***P < 0.001); *n* = 3; 1-tailed paired *t* test and presented as mean ± SEM.

Inflammation is a well-established component of the radiation injury response, representing the phase between acute DNA damage and later tissue remodeling and fibrosis (2, 3). We next asked whether p53-mediated DNA damage signaling in our model was accompanied by the production of pro-inflammatory cytokines. Across 3 independent donors, the “storm” of cytokines and chemokines known to respond to radiation-induced tissue injury (47–50) was increased in irradiated tissue compared with matched nonirradiated controls (Figure 4). Of these cytokines and chemokines, IL1β, CXCL1, CCL5, and CCL3 reached statistical significance, while the remaining cytokines trended toward upregulation with interdonor variability in the magnitude of response (Supplemental Figure 4). One donor displayed decreased levels of IL-6 and IL-8, consistent with prior reports of temporal and directional variability in these cytokines after ex vivo radiation (51). Together, these findings indicate that in addition to p53-associated DDR activation, irradiated ex vivo human skin features the pro-inflammatory cytokine cascade that characterizes the radiation injury response and is a known driver of radiation fibrosis (2, 3).

### Radiation triggers a profibrotic response involving EMT.

**Figure 5:**
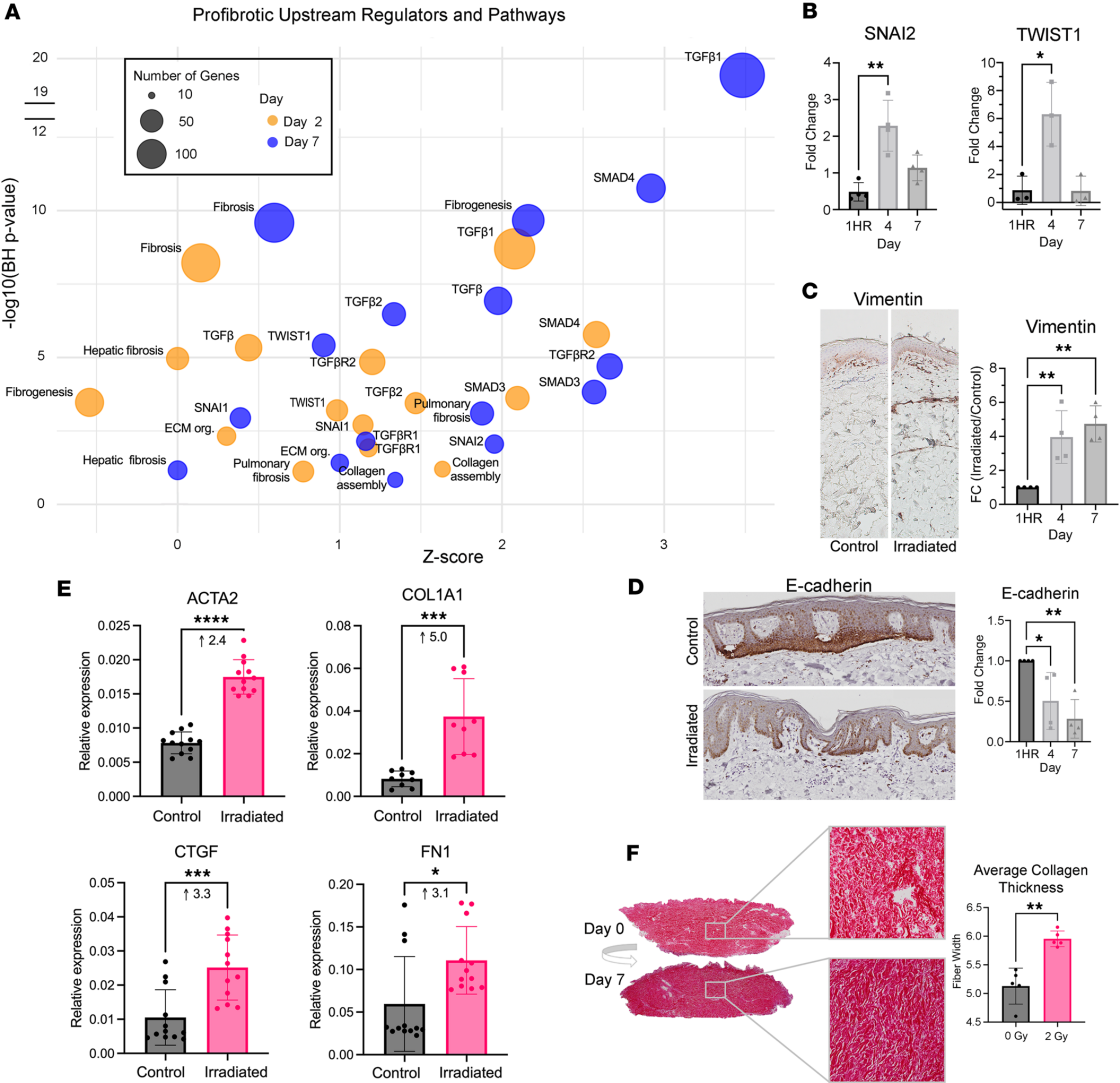
Induction of EMT and profibrotic genes in irradiated ex vivo skin. (**A**) Bubble plot depicting enrichment of profibrotic pathways, biological processes, and upstream regulators on day 2 (yellow) and day 7 (blue) after irradiation; *x* axis represents predicted activation *z* score, *y* axis shows the Benjamini-Hochberg–corrected –log_10_ *P* value, and bubble size reflects the number of overlapping genes. Data derived from IPA and plotted using ggplot2. (**B**) qPCR of EMT-inducing transcription in control (0 Gy) and 3.5 Gy irradiated skin on hour 1, days 4 and 7 after irradiation. Expression was normalized to 18S. (**C**) Representative IHC of vimentin in control and irradiated skin on day 4 after irradiation. (**D**) Representative IHC of E-cadherin in control and irradiated skin on day 4 after irradiation. (**E**) qPCR of profibrotic genes in control and 3.5 Gy irradiated skin on day 7 after irradiation. Expression was normalized to *GAPDH* and shown as relative expression (*n* = 9–12). (**F**) Picrosirius red staining of 2 Gy irradiated skin on day 0 and day 7 after irradiation with quantification of average collagen fiber thickness (*n* = 5). Asterisks indicate statistical significance (*P < 0.05, **P < 0.01, ***P < 0.001, ****P < 0.0001). *n* = 3 for **B**. *n* = 4 for **C** and **D**. Statistical analyses: 1-way ANOVA with Tukey’s correction for **B**–**D**; 2-tailed paired *t* tests for **E** and **F**. Total original magnification, ×10 (**C**), ×20 (**D** and **F**). Quantification presented as fold-change relative to control at each time point for **B**–**D**. All quantification for **B**–**F** presented as mean ± SD.

The final theme consistently maintained in the postirradiation RNA-seq datasets after p53 signaling was fibrosis. Specifically, a series of profibrotic upstream regulators and fibrogenic pathways were more significantly enriched and activated on day 7 versus day 2 after irradiation, suggesting a shift toward a more profibrotic environment in irradiated skin over time (Figure 5A). Among these, we noted predicted activation of EMT regulators Snail family transcriptional repressor 1 (*SNAI1*), Snail family transcriptional repressor 2 (*SNAI2*), and Twist-related protein 1 (*TWIST1*); qPCR analysis confirmed significant transcriptional upregulation of *TWIST1* and *SNAI2* in our model at 4 days after irradiation (Figure 5B). After skin injury, cutaneous EMT is marked by loss of E-cadherin and increased expression of vimentin as keratinocytes lose their cell-to-cell adhesion and acquire mesenchymal characteristics (52); therefore, we looked at these EMT mediators in irradiated ex vivo skin. We noted increased expression of the mesenchymal marker vimentin in irradiated dermis over time (Figure 5C). Conversely, there was reduced expression of E-cadherin most prominently in the basal keratinocytes of irradiated skin, and total protein levels of E-cadherin decreased over time after irradiation (Figure 5D and Supplemental Figure 5). Taken together, these results indicate a shift toward a more mesenchymal phenotype after irradiation.

### Induction of a TGFB1-mediated fibrotic phenotype in ex vivo skin after radiation.

Building on these early fibrotic signatures, our ex vivo model further demonstrated a robust *TGFB1*-mediated fibrotic phenotype. *TGFB1*, the master driver of tissue fibrosis (53), had the highest enrichment and activation scores among the profibrotic factors with predicted involvement in the radiation response in our ex vivo skin, with over 100 of its downstream gene targets overlapping with the postirradiation day 7 dataset (Figure 5A and Supplemental Table 1). To validate this in our model, we focused on the 4 genes previously identified as fibrotic *TGFB1* response genes in human ex vivo skin and linked to excessive skin scarring: α–smooth muscle actin (*ACTA2*), type 1 collagen (*COL1A1*), connective tissue growth factor (*CTGF*), and fibronectin 1 (*FN1*) (54, 55). We confirmed significant induction of all 4 genes after irradiation (Figure 5E), the highest of which was a 5.0-fold increase in *COL1A1*. These effects persisted across radiation doses of 2–6 Gy (not shown). Furthermore, quantitative analysis of Picrosirius red–stained collagen fibers (56) identified a marked increase in average collagen thickness on day 7 in irradiated skin compared with nonirradiated controls (Figure 5F).

### Biopsies of breast skin after irradiation contain p53, inflammatory, and profibrotic signals common to irradiated ex vivo skin.

**Figure 6:**
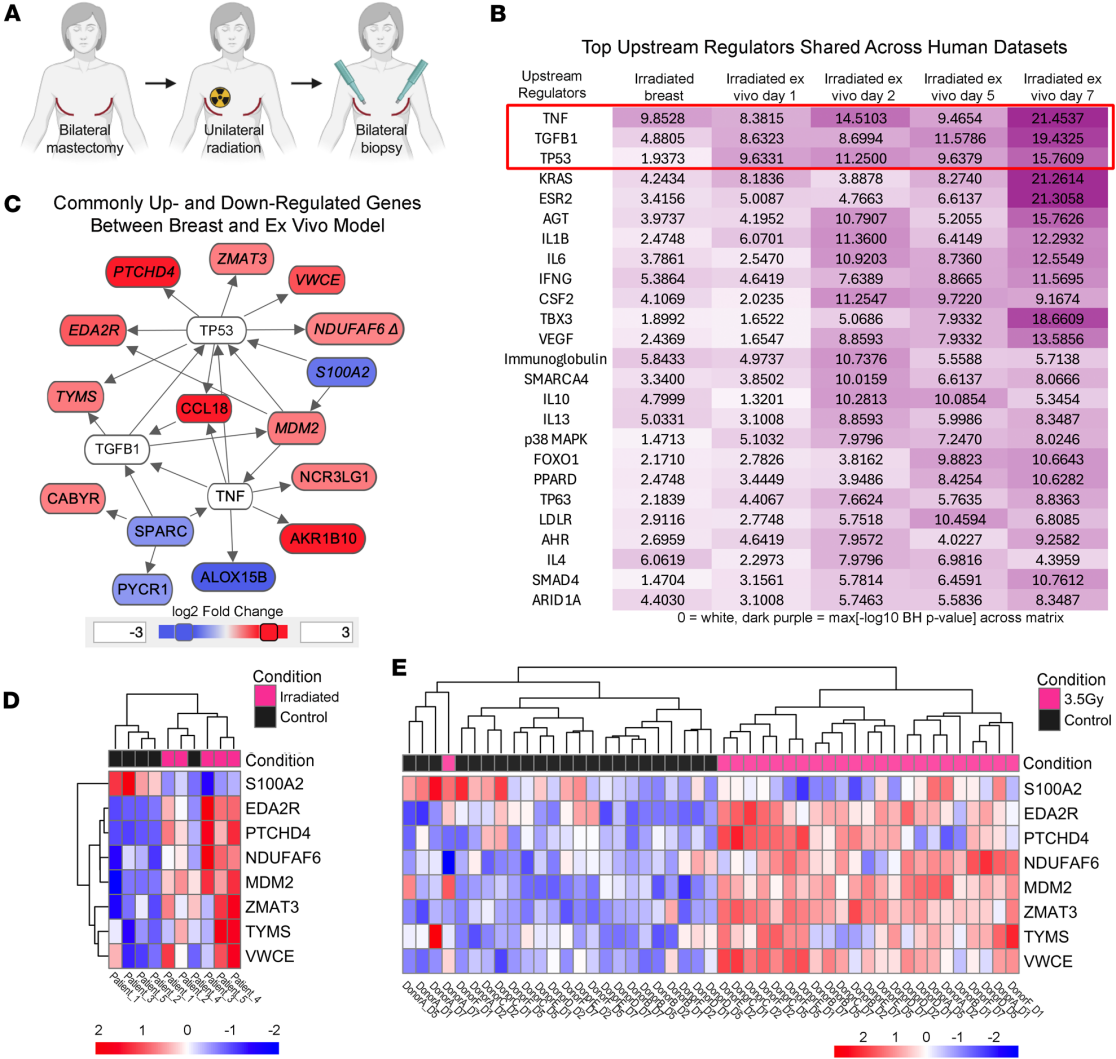
Correlation of ex vivo human skin radiation response with irradiated breast skin from patients after radiotherapy. (**A**) Skin biopsies of the irradiated breast skin and nonirradiated skin of the contralateral breast were obtained from *n* = 5 postmastectomy patients at the time of surgical reconstruction. Raw bulk RNA-seq profiles (GSE278183) were reprocessed and reanalyzed using IPA. (**B**) Top 25 enriched upstream regulators common to irradiated breast skin and ex vivo irradiated skin across days 1, 2, 5, and 7, ranked by sum of Benjamini-Hochberg enrichment *P* values across all time points. Matrix values represent –log_10_(Benjamini-Hochberg *P* value). Colored by conditional formatting (0 = white, max value = purple). (**C**) Network of the 15 genes overlapping between breast biopsies and at least 2 time points in the ex vivo irradiated skin. Red indicates commonly upregulated; blue is commonly downregulated across datasets. (**D**) Heatmap of 8 commonly regulated *TP53* genes across profiles from nonirradiated and irradiated breast skin (patients numbered 1–5). (**E**) Heatmap of the same *TP53* genes from **D** in human ex vivo skin. Data are log_2_-transformed counts per million (CPM) values. For **D** and **E**, red indicates increased expression; blue indicates decreased expression; annotation bars indicate the condition above the heatmap (pink = 3.5 Gy or irradiated; black = control). Heatmap was generated in RStudio using the pheatmap package.

To directly assess the clinical-translational relevance of the radiation response identified in our ex vivo model, we analyzed RNA-seq profiles of in vivo irradiated and nonirradiated contralateral breast skin biopsies harvested from 5 postmastectomy patients at the time of surgical reconstruction (GSE278183; Figure 6A). Using IPA’s Comparison Analysis tool, we identified substantial overlap in pathways and biological processes between irradiated patient breast tissue and ex vivo irradiated skin. Among the top 25 shared upstream regulators, *TNF*, *TGFB1*, and *TP53* ranked highest based on the sum of Benjamini-Hochberg–adjusted *P* values across time points (Figure 6B). Overlaying the gene datasets revealed 13 commonly regulated genes shared between the irradiated patient breast skin dataset and at least 2 time points of the irradiated ex vivo human skin dataset. These genes formed an interconnected network involving *TP53*, *TGFB*, the matricellular protein SPARC (57), and *TNF* (Figure 6C). Notably, commonly regulated *TP53* target genes in both irradiated patient breast biopsies and ex vivo skin included *MDM2* and *ZMAT3*, which we had validated in an expanded pool of ex vivo donors (Figures 2 and 3). The p53-radiation response observed across all 5 patients was consistent across ex vivo donors and sustained over time (Figure 6, D and E).

## Discussion

Although radiotherapy confers tremendous survival benefit for millions of patients with cancer, it also produces a spectrum of cutaneous side effects, including severe skin fibrosis that drastically impairs quality of life for patients for months to years after radiation exposure. Historically, methods to understand the cutaneous response to radiation have focused on UV radiation rather than ionizing radiation and have utilized primary cells (9, 10, 58), organoids (59–61), and animal models (27, 62, 63) that might not recapitulate the cutaneous radiotherapy toxicities observed in patients. As such, an incomplete understanding of the mechanisms of radiation-induced skin injury and its evolution to fibrosis in patients has hindered the development of efficacious therapies. To address this gap, we developed a human ex vivo skin model that demonstrates a reproducible temporal and dose-dependent radiation injury response, providing insights into the early evolution of radiation-induced skin injury and fibrosis. Importantly, our findings demonstrate that this model recapitulates key features of the in vivo radiation injury response observed in skin biopsies from postradiotherapy surgical reconstruction patients, establishing its clinical-translational relevance.

We found that radiation rapidly initiates a DDR centered on p53 and its downstream transcriptional program in our model, successfully recapitulating the acute radiation response observed in many organs and tissues in vivo. This pathway regulates cell fate decisions by inducing cell cycle arrest and senescence to allow DNA repair or by initiating apoptosis of irreparably damaged cells. The robust and expected activation of this fundamental cellular response in our ex vivo model further validates its ability to mimic the acute effects of radiation on human skin tissue. Our model also captured elements of the “cytokine storm” produced by injured keratinocytes and fibroblasts as part of the inflammatory phase of the cutaneous radiation response (Figure 4). IL-1, IL-6, and TNF-family cytokines are central mediators of the early response and promote downstream chemokine production, including CXCL1, CXCL8 (IL-8), and CCL chemokines, thereby amplifying local inflammatory signaling and immune cell recruitment (64–68). A coordinated pattern of upregulation was observed for most cytokines at 7 days after irradiation in the ex vivo skin, though the magnitude of induction varied by donor (Supplemental Figure 4). Along with p53 signaling and pro-inflammatory cytokines, we identified a strong profibrotic radiation response involving EMT and TGF-β1 that mimics global phenotypic features of skin fibrosis (Figure 5). These results offer substantial evidence that our human ex vivo skin model successfully characterizes the multifaceted and dynamic response to radiation-induced injury — p53-DDR, pro-inflammatory cytokine production, and the progression toward fibrosis — making it a powerful and clinically relevant surrogate for investigating RISF and targeted therapeutic interventions.

In the clinical setting, radiotherapy is delivered in fractionated form rather than as a single dose to reduce healthy tissue toxicity (4, 8). For example, the conventional breast cancer radiotherapy protocol is 50 Gy delivered in 25 fractions of 2 Gy daily over 5 weeks (69, 70); single doses above 6–8 Gy are uncommon due to risk of skin and subcutaneous tissue necrosis (71, 72). The skin explants have a limited window of viability within which to deliver multiple fractions, so in this study we initially utilized the single dose of 3.5 Gy to successfully demonstrate the radiation injury response and subsequently explored the dose-response effect using the 6 Gy treatment. Given that the ex vivo skin’s viability was successfully maintained with these single-dose treatments, future studies are planned to deliver higher total doses in smaller fractions, to better recapitulate the clinical radiotherapy regimens, further enhancing the model’s translational relevance.

We further demonstrated the clinical-translational applicability of our model by identifying the consistency in the p53-DDR, inflammation, and fibrotic responses between the ex vivo skin and breast skin from patients after radiotherapy, as represented by the conservation of *TP53*, *TNF*, and *TGFB1* regulatory signaling nodes (Figure 6, B and C). *TNF*, the top shared transcriptional upstream regulator in the irradiated breast skin and ex vivo model, encodes TNF-α protein whose levels increased in ex vivo skin from all 3 donors after irradiation while remaining low to undetectable in the nonirradiated controls (Figure 4 and Supplemental Figure 4). Though this trend did not reach statistical significance, the cytokines lying downstream of TNF-α (e.g., CXCL1, CCL5) were significantly elevated in the irradiated ex vivo skin. Moreover, it is well established that transcriptional evidence of pathway activation may not translate into proportional changes in steady-state protein abundance; even in patients with breast cancer, studies conflict as to whether TNF-α cytokine levels are significantly elevated after radiotherapy (73) despite the strong transcriptional response. We therefore interpret the concordant TNF-related findings between ex vivo and patient skin as evidence that the TNF axis is similarly engaged in both settings.

Fibrosis is a progressive process of abnormal matrix deposition that evolves after unresolved inflammation in the context of an ineffective healing response, and radiation injury exacerbates this process by further fueling the pro-inflammatory response and collagen production (3). Although the canonical role of p53 in regulating DNA damage/radiation injury is known (24, 25, 30), its role in driving radiation-induced fibrosis has not been explored. Recent studies have supported a causal role for p53 in organ fibrosis and have advocated for therapeutic targeting of p53 to mitigate hepatic, pulmonary, renal, and cardiac fibrosis (74). In this regard, p53 plays a mechanistic role in many biological processes that aggravate fibrosis, including TGF-β signaling, EMT, and apoptosis (75–78). P53 also induces senescence as part of the cellular stress response, driving the senescence-associated secretory phenotype and secretion of pro-inflammatory cytokines and matrix-modulating factors that notably include TGF-β1 itself (79–81). In the context of radiation injury, this senescence-associated remodeling can exacerbate fibrotic responses, especially when senescent cells persist and evade immune clearance (80, 81). As such, sustained p53 signaling may be a critical driver in the transition from initial radiation injury to chronic fibrosis in the skin, making it a prime candidate for therapeutic targeting. In this regard, our data conceptually support leveraging activities of radiation-induced p53 regulators such as MDM2 and miR-34a to fine-tune the p53 response such that it acts to repair damaged DNA and injured skin, then shuts off before driving remodeling to the point of debilitating fibrosis. For example, miR-34a suppresses EMT by targeting EMT-inducing transcription factors (82); thus, targeted modulation of its activity may mitigate the early profibrotic response. Similarly, MDM2 inhibitors such as nutlin-3a and navtemadlin have demonstrated antifibrotic effects in preclinical and clinical trial settings (83, 84) and might be repurposed to manage cutaneous radiation toxicities. Moreover, because TGF-β1 signaling, EMT, and matrix remodeling are conserved profibrotic pathways across organs (85–87), the programs captured in our irradiated skin explants likely mirror key elements of radiation-induced fibrogenesis in other tissues. Given that many of the transcriptional and signaling responses we identify converge on generic wound-healing and matrix-remodeling pathways, this ex vivo RISF model may reflect core fibrogenic processes shared across organ systems. As such, it provides a valuable human platform for generating mechanistic insight into fibrogenesis and for testing candidate antifibrotic strategies with relevance to radiation-associated fibrosis beyond the skin.

Our study has several limitations. Ex vivo skin is detached from the blood supply and does not capture the dynamic recruitment of immune cells and inflammatory mediators to the site of radiation injury. However, we have previously shown that resident immune cells, including Langerhans cells and γδ T cells, are maintained in the ex vivo skin for at least 7 days using our culture conditions (88–90). Moreover, skin injury triggers the innate immune functions of keratinocytes and fibroblasts to release pro-inflammatory cytokines, produce antimicrobial peptides, and activate skin-resident immune cells. Indeed, the ex vivo skin not only responded to radiation injury with upregulation of cytokines, but also recapitulated pro-inflammatory signals seen in the in vivo irradiated breast skin, despite its lack of blood supply (Figure 4 and Figure 6B). Additionally, although cell populations within the skin exhibit differential radiosensitivity, bulk transcriptomic data make it difficult to resolve cell-specific signatures of radiation injury and fibrosis, potentially obscuring key radiation response pathways. Future studies leveraging spatial single-cell technologies are needed to further explore the cell-specific radiation responses, with the goal of targeting the cells driving a sustained pro-inflammatory and profibrotic response to mitigate skin radiation injury. Variability in responses among human skin donors was another anticipated limitation that was mitigated by the study design of paired control and irradiated skin from each donor as well as by increasing biological replicates (donors). Finally, although our findings suggest the connection between sustained p53-mediated DDR signaling and the profibrotic skin radiation response, mechanistic studies are needed to validate a causal relationship.

Regarding therapies for RISF, it is important to note that the p53-mediated effects are critically needed to induce death of cancer cells within the tumor being targeted; as such, topical (rather than systemic) formulations are likely best suited to mitigate and treat cutaneous radiation toxicities. This consideration is particularly relevant given that approximately half of cancers harbor TP53 mutations (91), underscoring the continued importance of p53-dependent pathways for tumor control, even as these same pathways contribute to radiation-driven fibrogenesis in normal tissue. Transient p53 activity is also temporarily needed to repair skin cells damaged by radiation, to mitigate the development of secondary skin cancers. Moreover, varying radiation doses and dose rates may differentially engage p53-damage responses that permit injury resolution versus those that tip the balance toward persistent fibrosis, analogous to the bleomycin lung injury model in which a single dose induces self-limited inflammation, whereas repeated dosing results in irreversible fibrosis (92–94). Comparing molecular and transcriptional activation signatures across a gradient of radiation doses in our model may help identify unique profibrotic elements that are specifically required to shift from an initial p53-DDR/inflammatory response with potential for resolution to a state of persistent fibrosis. Taken together, therapies will need be timed to the inflection point at which p53 signaling exhausts the skin-reparative effects and shifts to drive excess pro-inflammatory and profibrotic signaling. Future studies aimed at identifying this critical time window and developing therapeutic strategies to precisely modulate these fibrosis-shifting elements after radiation injury are needed to prevent progression to severe irreversible skin fibrosis. Furthermore, identification of early tissue biomarkers that best capture evolving RISF (as opposed to transient radiation injury) could serve as companion diagnostics to predict which patients are likely to progress to severe fibrosis, thereby enabling earlier intervention and alleviating the suffering of patients surviving cancer who are experiencing impaired quality of life from cutaneous radiation toxicities.

## Methods

### Sex as a biological variable.

Human ex vivo skin was obtained from adult panniculectomy donors, consisting of predominantly samples from women (*n* = 11) and a single sample from a man (*n* = 1). Sex was not included as a variable in this study due to the sample size for male donors, which was insufficient to support sex-stratified analyses. Given that our primary endpoints relate to acute ex vivo responses of full-thickness skin under controlled culture conditions, we expect the main findings to be broadly relevant across sexes.

### Human ex vivo RISF culture conditions.

Panniculectomy specimens that were less than 6 in^2^, had visible striae involving more than 50% of the specimen, and/or had a severely thinned dermis, giving a more translucent appearance, were excluded from this study. After excision in the operating room, subcutaneous fat was removed from the skin samples, placed into sterile cups, and transferred into the biosafety cabinet. Each skin sample was examined in a 150 mm × 25 mm cell culture plate containing sterile PBS to assess tissue quality and ensure the removal of any remaining subcutaneous fat. Skin was then sectioned into approximately 2-in by 2-in pieces and subjected to a sequential decontamination process, via washes in 5 consecutive 50 mL conical tubes containing 70% ethanol followed by 3 sterile PBS washes. After the final PBS wash, decontaminated skin sections were placed dermis-side down into fresh 150 mm × 25 mm tissue culture plates containing prewarmed supplemented culture media. Media was prepared using 1× DMEM with GlutaMAX (Gibco, 10566016) as the base. To this, 10% non–heat-inactivated FBS (Cytiva HyClone, SH30071.03), 1% antibiotic-antimycotic (Gibco, 15240-062), and 1% sodium pyruvate (Gibco, 11360-070) were added. Media was thoroughly mixed by gentle inversion and vacuum-filtered using a Bottle-Top Vacuum Filtration System with a 0.2 μm PES membrane (VWR, 514-0332) to ensure sterility. Each tissue culture plate received a sufficient amount of media to fully submerge the dermis without covering the epidermis and was placed on ice prior to further experimental procedures.

### Human ex vivo RISF skin model and sample collection.

Human skin specimens were irradiated with single doses of 3.5 or 6 Gy delivered by an Xstrahl cabinet irradiator, model RS225, with the following parameters: 190 kV and 10 mA with 0.5 mm Cu filter for 3 minutes and 42 seconds for 3.5 Gy or 6 minutes and 18 seconds for 6 Gy. Irradiated skin was maintained with donor-matched (nonirradiated) control skin at an air-liquid interface at 37°C**/**5% CO_2_ for up to 7 days using a modified protocol from Mouawad et al. (54). Control and irradiated skin were collected at the following time points: immediate (30–60 min) and days 1, 2, 4, 5, and 7 after irradiation. At each time point, tissue samples were placed in RNAlater Stabilization solution (Thermo Fischer Scientific, AM7020) for RNA isolation, snap-frozen for protein isolation, and fixed in 10% formalin for subsequent paraffin embedding.

### RNA isolation and quality control.

RNAlater-preserved samples were maintained on ice while being dissected and weighed (<30 mg). Samples were then finely chopped with a scalpel prior to being placed in tubes with RLT-βME with 3.0 mm zirconium beads (Benchmark Scientific, Inc., D10320-30). Samples then underwent 3–4 rounds of homogenization at 400 speed for 30 seconds with 1 minute rest between rounds in a BEADBug6 (Benchmark Scientific, Inc.) homogenizer. Lysates were centrifuged at 16,000*g* for 3 minutes, and the supernatant was thoroughly mixed with an equal volume of 70% ethanol. Total RNA was then isolated using QIAGEN’s RNeasy Mini kit following the manufacturer’s protocol. RNA concentration and purity were determined via NanoDrop spectrophotometry (NanoDrop 2000) and RNA integrity was assessed via QIAxcel (QIAGEN) to determine the RNA Integrity Score (RIS). Samples with a 260/230 greater than 1.6 and RIS greater than 4.5 were used for downstream studies.

### Real-time qPCR.

First, 1.0 μg of total RNA from ex vivo samples were reverse-transcribed using QuantiTect Reverse Transcription kit (QIAGEN) to generated cDNA, and real-time qPCR was performed in triplicate using PerfeCTa SYBR Green Supermix (QuantaBio) and the Bio-Rad CFX Connect system. The relative expression of target genes was normalized to housekeeping genes *18S*, *ARPC2*, or *GAPDH* (or a combination thereof) based on the requirements of each qPCR analysis. For miRNA (*miR-34a-5p*) qPCR, 50–75 ng of total RNA from ex vivo samples was reverse-transcribed and enriched for miRNAs using microScript microRNA cDNA Synthesis kit (Norgen Biotek). Real-time qPCR was performed as stated above with relative expression normalized to levels of SNORD48. Primer sequences can be found in Supplemental Table 2.

### RNA-sequencing.

RNA from 6 donors underwent ribosomal RNA depletion with the resulting mRNA used for library preparation and subsequent bulk RNA-seq on the Illumina NovaSeq X Plus at Azenta Life Sciences. Library preparation was performed by Azenta Life Sciences using standard RNA-seq protocol, yielding an average of 25 million to 30 million paired-end reads per sample. Quality control of the raw sequencing reads was initially assessed using FastQC (https://www.bioinformatics.babraham.ac.uk/projects/fastqc/). Adapter sequences and low-quality bases were trimmed using TrimGalore in paired-end mode (95) (https://www.bioinformatics.babraham.ac.uk/projects/trim_galore/). Trimmed reads were then aligned to the GRCh38 human genome using the STAR aligner (version 2.7.10a) with gene counts being quantified from GENCODE Release 44 gene annotations (96). Raw data files and the associated count matrix are available at NCBI’s Gene Expression Omnibus (GEO; GSE297035). Alignment metrics were generated using GATK (version 4.3.0.0) CollectRnaSeqMetrics tool, with the hg38 reference file for annotation (97). MultiQC visually summarized all previous steps (98). Batch correction was performed using ComBat (version, 3.54.0; ref. 99) prior to paired differential expression via edgeR (99, 100). Analyses were restricted to protein-coding genes, and genes were considered differentially expressed if they met a nominal *P* value of 0.05 or less and log_2_ fold-change of ±0.5 or greater. This threshold was chosen to balance specificity and sensitivity in identifying time-dependent gene expression changes across donors and to enable robust downstream exploratory pathway analysis using IPA (QIAGEN), which applies Benjamini-Hochberg correction to account for multiple testing.

### Bioinformatics and pathway analyses.

All RNA-seq data processing and visualization were performed in R (2024.12.1+563) (101). Visualizations were generated using packages including edgeR, tidyverse (e.g., dplyr, ggplot2, tidyr) (102), RcolorBrewer (103), pheatmap (104), gplots (105), and gridExtra (106). Heatmaps were scaled by row and clustered using Euclidean distance unless otherwise noted. The bubble plot was created with ggplot2.

Differentially expressed genes identified at each time point (3.5 Gy vs. control) underwent IPA Core Analysis, which yielded enrichment *P* values and predicted activation (*z* scores) for canonical pathways, biological processes, and upstream regulators. Enrichment *P* values were corrected for multiple testing using the Benjamini-Hochberg method, and results with Benjamini-Hochberg–adjusted *P* of 0.05 or less and an absolute *z* score of 2 or greater were considered significant.

### Total protein isolation and Western blotting.

For Western blotting, 15–25 mg of snap-frozen tissue was lysed in RIPA buffer containing 150 mM NaCl, 1% NP-40/Triton X-100, 0.5% sodium deoxycholate, 0.1% SDS, and 50 mM Tris (pH 8.0), supplemented with Protease and Phosphatase Inhibitor Cocktail (Benchmark Scientific, Inc.). Tissue homogenization was performed using 3 mm triple-pure zirconium beads (Benchmark Scientific, Inc.) in a BeadBug microtube homogenizer (Benchmark Scientific, Inc.). Protein concentration was quantified using the Qubit Protein Assay kit (Thermo Fisher Scientific, Q33211) and the Qubit 4 Fluorometer (Thermo Fisher Scientific).

Equal amounts of total protein were resolved by SDS-PAGE using 4%–20% Criterion TGX precast gels (Bio-Rad), followed by transfer to PVDF membranes (Bio-Rad). Membranes were blocked in TBST (TBS + 0.1% Tween-20) containing 5% nonfat dry milk for 1 hour at room temperature and then incubated overnight at 4°C with primary antibodies diluted in blocking buffer. Membranes were washed in TBST and incubated with HRP-conjugated secondary antibodies for 1 hour at room temperature. Bands were visualized using chemiluminescent substrate and imaged using a Bio-Rad ChemiDoc MP Imaging system. Band intensities were quantified using ImageJ (NIH) by background-subtracted densitometry and normalized to corresponding loading controls. Antibodies, including vendor, catalog number, and dilution, are listed in Supplemental Table 3.

Cytokine profiling was performed on whole-tissue lysates from irradiated and matched control ex vivo human skin harvested on day 7 after irradiation using the Proteome Profiler Human Cytokine Array kit (R&D Systems, ARY005B), according to the manufacturer’s instructions. Tissue processing and protein extraction followed the general workflow described above, and equal amounts of protein were applied to each membrane. Cytokine signals were detected by chemiluminescence and quantified using ImageJ (NIH). Background subtraction was performed using negative control regions on each membrane, duplicate spots for each cytokine were averaged, and signal intensities were normalized to the internal reference spots provided on the array. Statistical comparisons between irradiated and control samples were performed using 1-tailed paired *t* tests.

### Tissue immunostaining.

FFPE tissues were sectioned at 5–6 μm using a microtome, deparaffinized in xylene (EMD Millipore), and rehydrated through a graded ethanol series. Antigen retrieval was performed by incubating slides in sodium citrate buffer (10 mM sodium citrate, 0.05% Tween-20, pH 6.0) at 95°C for 30–45 minutes, followed by a 20-minute cool-down at room temperature for IHC and an ice bath for IF. Endogenous peroxidase activity was quenched (for IHC only) using 0.3% hydrogen peroxide in methanol. Slides were washed with TBST and blocked for 1 hour at room temperature with 2% normal goat serum (Sigma-Aldrich) in TBST or 10 minutes with Background Punisher (BIOCARE Medical).

Primary antibodies were diluted in SignalStain Antibody Diluent (Cell Signaling Technology) for IHC and 5% BSA/TBST for IF, and then applied overnight at 4°C. For IHC, detection was performed using SignalStain Boost IHC Detection reagent (HRP, Rabbit) and developed using SignalStain DAB Chromogen (Cell Signaling Technology). Slides were counterstained with Harris hematoxylin (Leica Microsystems), dehydrated through ethanol and xylene, and mounted using Cytoseal XYL (Epredia, 8310-4). For IF, after incubation with primary antibodies and washing, sections were incubated with fluorophore-conjugated secondary antibodies (Alexa Fluor 488 or 594; Invitrogen) for 1 hour at room temperature. Nuclei were counterstained with DAPI, and slides were mounted with antifade mounting medium. IHC slides were imaged using the Olympus VS200 Slide Scanner and analyzed in QuPath (v0.5.1) for percentage positive area, staining intensity, and area fraction. IF slides were imaged using the Olympus DP74 confocal microscope digital camera.

All antibodies used, including vendor, catalog number, and dilution, are listed in Supplemental Table 3.

### Quantification of tissue immunostains.

Quantification of IHC staining (E-cadherin and vimentin) was performed using QuPath (v0.5.1) as previously described (107). Positive staining was normalized to the percentage positive area per 100,000 μm² tissue section. Quantification of IF staining (γH2AX and MDM2) used the percentage of positive nuclear staining as manually scored by 3 independent blinded observers, calculated as (no. positive nuclei/no. total DAPI-stained nuclei) × 100, and the average values were used for analysis.

### Assessment of dermal collagen thickness.

Picrosirius red staining was performed using a Picrosirius red stain kit (Polysciences, Inc., 24901) to visualize and quantify collagen types I and III in paraffin-embedded tissue sections. Sections were deparaffinized in xylene and rehydrated through a graded ethanol series, finishing with distilled water. Nuclei were stained with Weigert’s hematoxylin for 8 minutes, followed by thorough rinsing in distilled water. Slides were then stained in Solution A for 2 minutes, rinsed in distilled water, and subsequently stained in Solution B for 60 minutes. After staining, sections were placed in Solution C for 2 minutes, followed by a brief rinse in 70% ethanol for 45 seconds. Slides were then dehydrated through graded ethanol, cleared in xylene, and mounted using a suitable mounting medium. Stained sections were imaged using a polarized light microscope with multiple fields of view being imaged to cover representative areas of each tissue section. Collagen fiber analysis was conducted using CT-FIRE software, as previously described (56), where images of Picrosirius red–stained sections were uploaded to CT-FIRE software for segmentation and quantification of collagen fibers, providing metrics on collagen fiber thickness.

### Statistics.

Data were analyzed using GraphPad Prism software and R (version 2024.12.1+563). For RNA-seq analyses, differential gene expression was assessed using edgeR with a paired design to account for matched control and irradiated samples from the same donor. *P* values were adjusted for multiple testing using the Benjamini-Hochberg method, and genes with an adjusted *P* value of 0.05 or less and an absolute log_2_ fold-change of ±0.5 or greater were considered differentially expressed.

For qPCR experiments, relative gene expression was calculated using the ΔΔCt method. At each time point, irradiated samples were normalized to corresponding controls to determine fold change. Statistical testing was performed using a mixed-effects model with a repeated-measures design, followed by Tukey’s post hoc test for multiple comparisons within each time point (GraphPad Prism).

Where indicated, comparisons between 2 groups were performed using paired Student’s *t* tests (1 or 2 tailed, as specified in the figure legends). For experiments involving more than 2 groups, 1-way or 2-way ANOVA was performed as appropriate, with Tukey’s correction for multiple comparisons, as specified in the figure legends. A *P* value less than 0.05 was considered statistically significant.

### Study approval.

Human skin was obtained from patients undergoing elective panniculectomy procedures under a protocol approved by the University of Miami IRB (IRB 20070922). Age, sex, and skin tone (e.g., light, medium, or dark) of each patient was recorded. Study participants provided written informed consent prior to surgery and collection of skin.

### Data availability.

RNA-seq data (raw files and count matrix) have been deposited to NCBI’s GEO (GSE297035). Values for all data points in graphs are reported in the Supporting Data Values file.

## Author contributions

RCS, MTC, IP, and AJG conceptualized the study. GD, LS, RCS, SRT, AJG, CD, and SMB developed the methodology. CD, SMB, GD, HP, LS, and JAKJ conducted experimentation and investigation. CD, SMB, GD, HP, RCS, and AJG generated visualizations. RCS, MTC, and IP acquired funding. RCS, AJG, MTC, IP, and SRT supervised the project. CD, SMB, and RCS wrote the original draft of the manuscript. CD, SMB, GD, HP, LS, JAKJ, SRT, IP, MTC, AJG, and RCS reviewed and edited the manuscript. The order of co–first authorship was determined based on primary responsibility for advancing the manuscript to completion.

## Conflict of interest

The authors have declared that no conflict of interest exists.

## Funding support

This work is the result of NIH funding, in whole or in part, and is subject to the NIH Public Access Policy. Through acceptance of this federal funding, the NIH has been given a right to make the work publicly available in PubMed Central.

- Dermatology Foundation Physician Scientist Career Development Award (to RCS).
- NIH grant R61/R33 DK131897.
- NIH grant R01AR083385.

## Supplementary Material

Supplemental data

Unedited blot and gel images

Supporting data values

## Version 1 03/19/2026

In-Press Preview

## Version 2 07/08/2026

Electronic publication
