# Supplementary material for: A human ex vivo model of radiation-induced skin injury recapitulates p53-driven profibrotic response to radiotherapy
Source: JCI Insight. 2026 Mar 19;11(13):e198692. doi: 10.1172/jci.insight.198692 (PMC13460817; doi:10.1172/jci.insight.198692)
Supplement: Supplementary file 1 [file jciinsight-11-198692-s126.pdf]

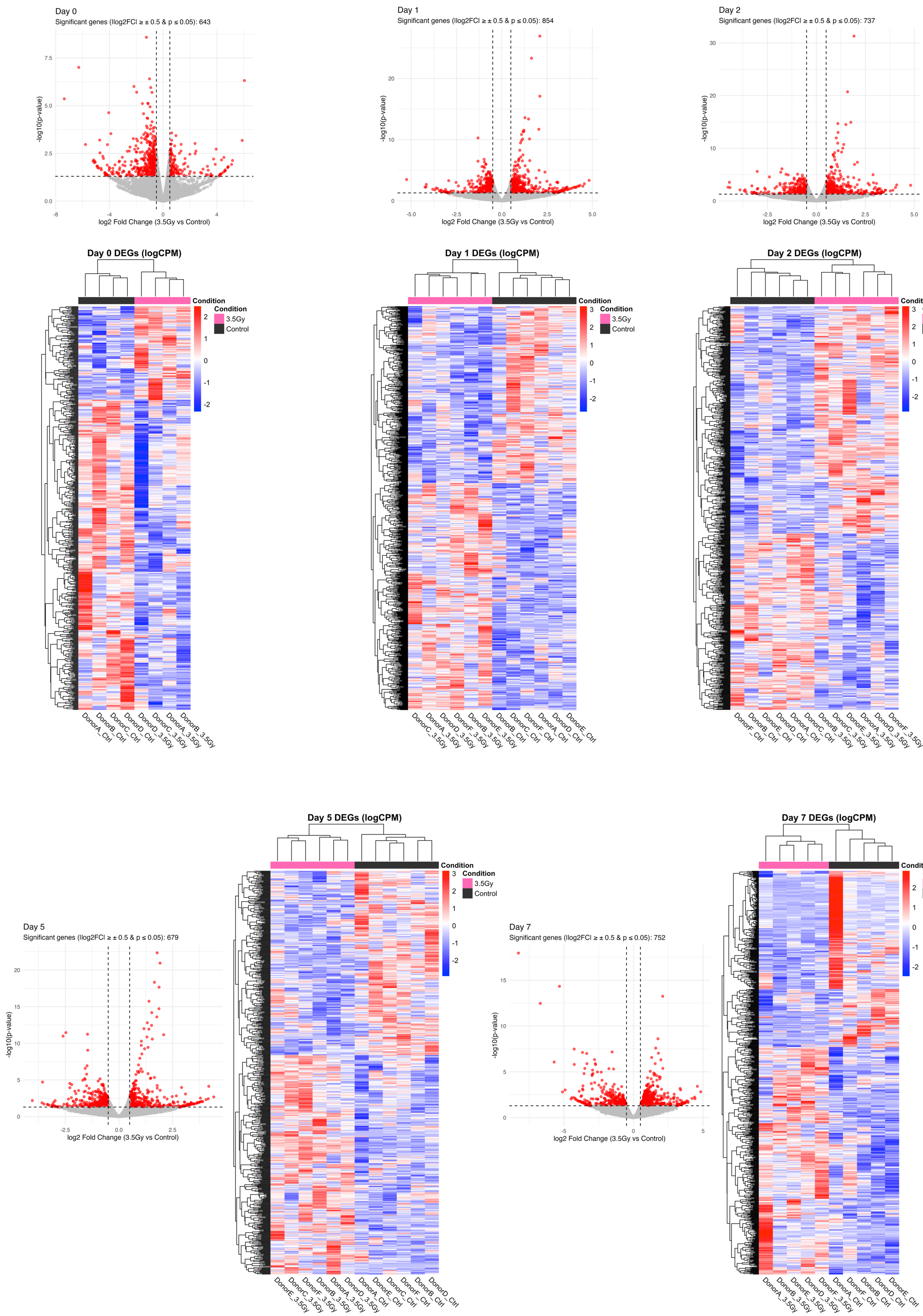

Day 0
Significant genes (|log2FC| ≥ ± 0.5 & p ≤ 0.05): 643
Day 1
Significant genes (|log2FC| ≥ ± 0.5 & p ≤ 0.05): 854
Day 2
Significant genes (|log2FC| ≥ ± 0.5 & p ≤ 0.05): 737
-log10(p-value)
log2 Fold Change (3.5Gy vs Control)
Day 0 DEGs (logCPM)
Day 1 DEGs (logCPM)
Day 2 DEGs (logCPM)
Condition
3.5Gy
Control
Day 5
Significant genes (|log2FC| ≥ ± 0.5 & p ≤ 0.05): 679
Day 5 DEGs (logCPM)
Day 7
Significant genes (|log2FC| ≥ ± 0.5 & p ≤ 0.05): 752
Day 7 DEGs (logCPM)

Supplemental Figure 1. Differentially expressed genes in irradiated vs. control skin by donor across time points. Volcano plots display the results of differential gene expression analysis for each time point following 3.5 Gy-radiation exposure compared to control. Each point represents a gene, with the x-axis showing the log_2FC and y-axis showing the $-log_{10}$ p-value. Genes highlighted in red are those that met the significance thresholds of an absolute $log_2FC \geq \pm 0.5$ and p-value ≤ 0.05. The number of significant genes (meeting both thresholds) is indicated within each volcano plot. Heatmaps are visualizing the expression patterns of these significant genes across individual donors at each corresponding time point. Each row in the heatmap represents a gene, and each column represents an individual sample from a unique donor at that time point. The color intensity corresponds to the gene's expression level (red = increased, blue = decreased).

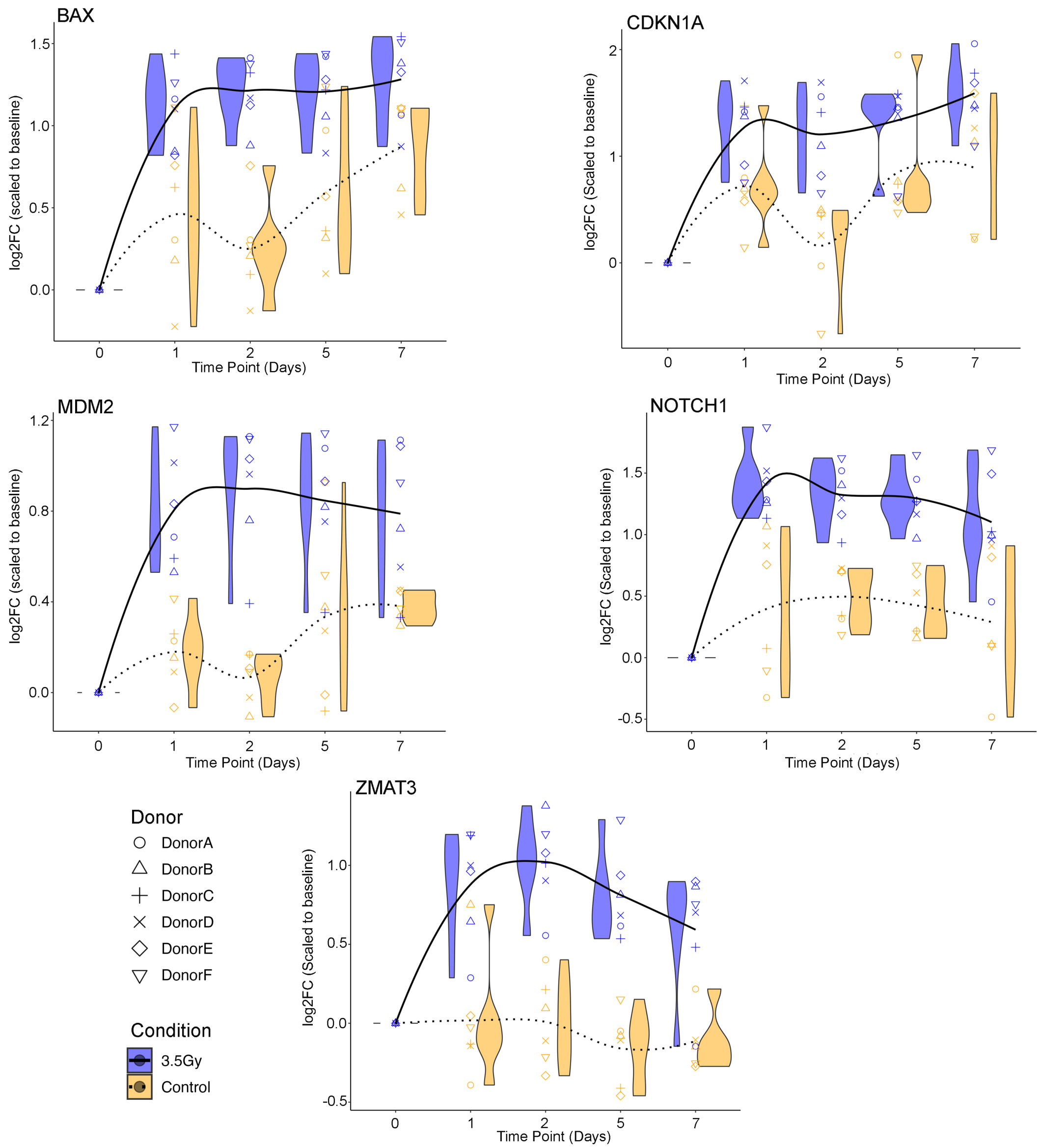

Supplemental Figure 2. Trajectories of p53 target genes over time following irradiation. Trajectory plots illustrate the temporal expression patterns of specific genes following exposure to 3.5 Gy-irradiation (blue) or control (yellow). Each point represents the $\log_2$ fold change ($\log_2$FC) in gene expression after being scaled to the earliest time point (day 0). Individual donors are distinguished by unique symbols. The x-axis represents time, and the y-axis displays the $\log_2$FC. Violin plots are overlaid at each time point to show the distribution of $\log_2$FC values across all donors within each treatment group. Solid and dotted lines represent the mean $\log_2$FC across all donors within the 3.5 Gy and control groups, respectively, at each time point.

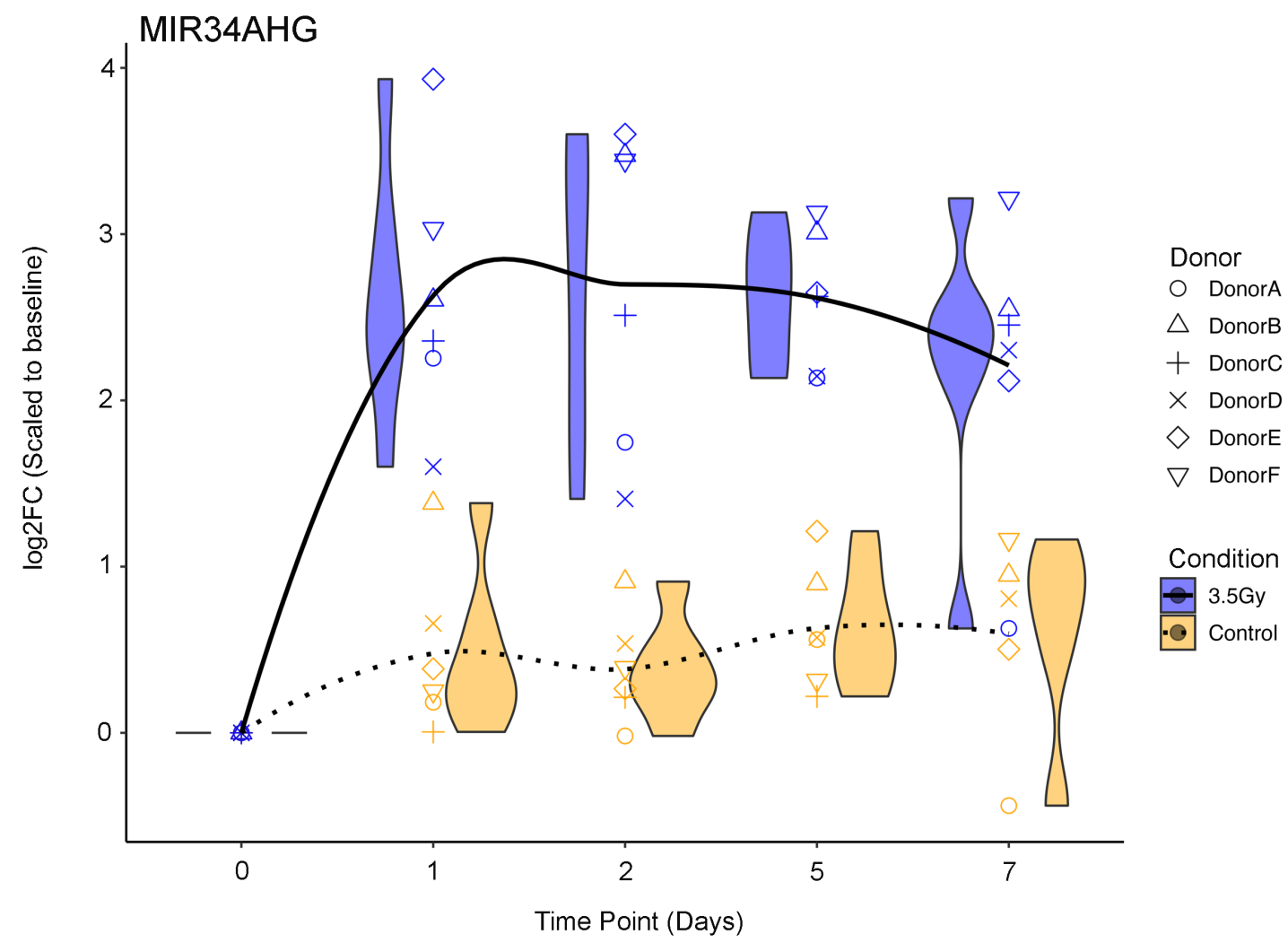

Supplemental Figure 3. Trajectory of MIR34AHG over time following irradiation. Trajectory plot illustrating the temporal expression patterns of MIR34AHG following exposure to 3.5 Gy-irradiation (blue) or control (yellow). Each point represents the log_2 fold change (log_2FC) in gene expression after being scaled to the earliest time point (day 0). Individual donors are distinguished by unique symbols. The x-axis represents time, and the y-axis displays the log_2FC. Violin plots are overlaid at each time point to show the distribution of log_2FC values across all donors within each treatment group. Solid and dotted lines represent the mean log_2FC across all donors within the 3.5 Gy and control groups, respectively, at each time point

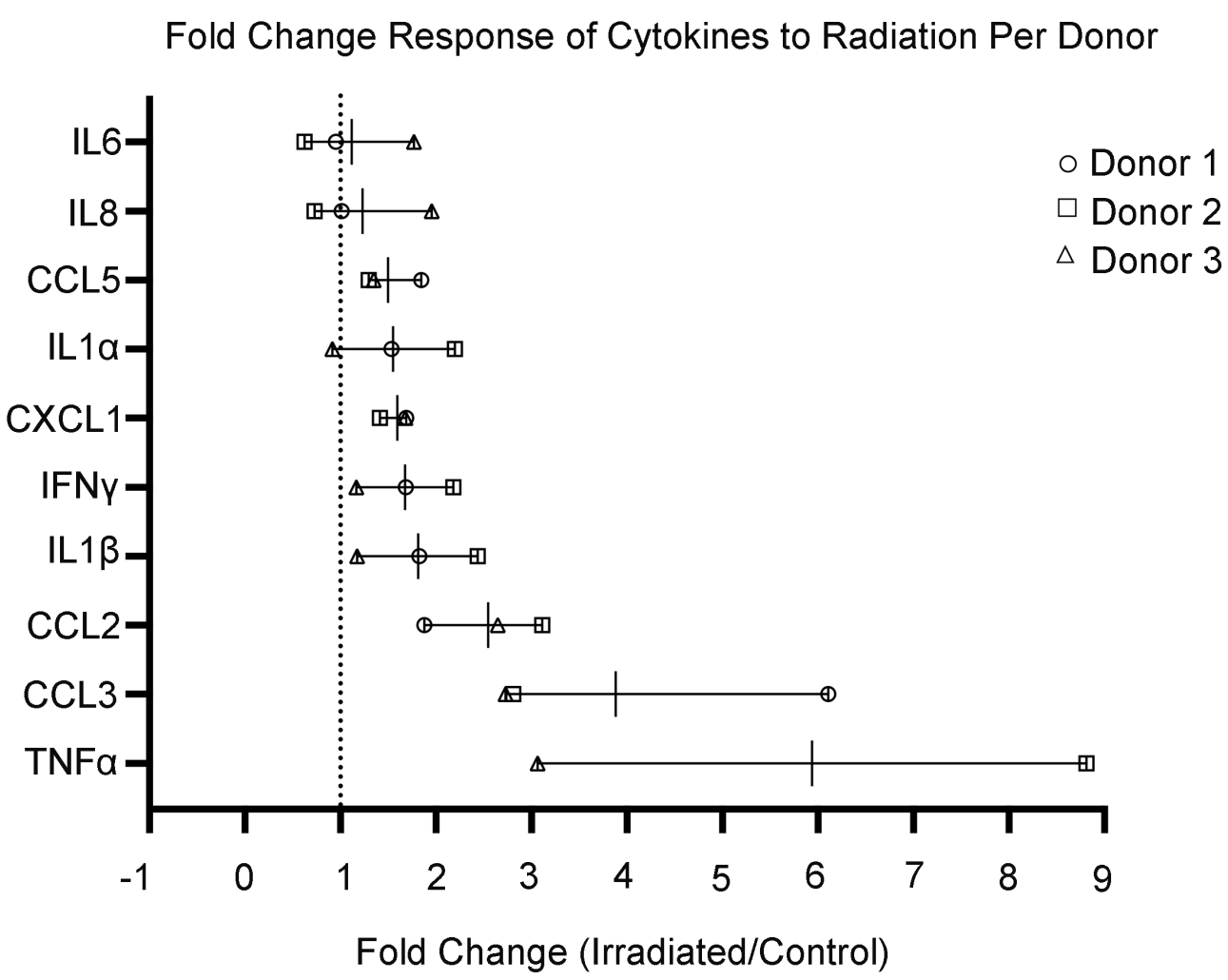

Supplemental Figure 4. Upregulation of pro-inflammatory cytokines across donors post-irradiation. Aligned dot plot depicting fold change (irradiated relative to matched control) for pro-inflammatory cytokines (corresponding to Figure 4) across individual donors. Each point represents the fold change for a given cytokine in a single donor. Values > 1 indicate higher expression in irradiated skin compared to control. Fold change in TNFα for Donor 1 could not be calculated due to undetectable levels in control skin.

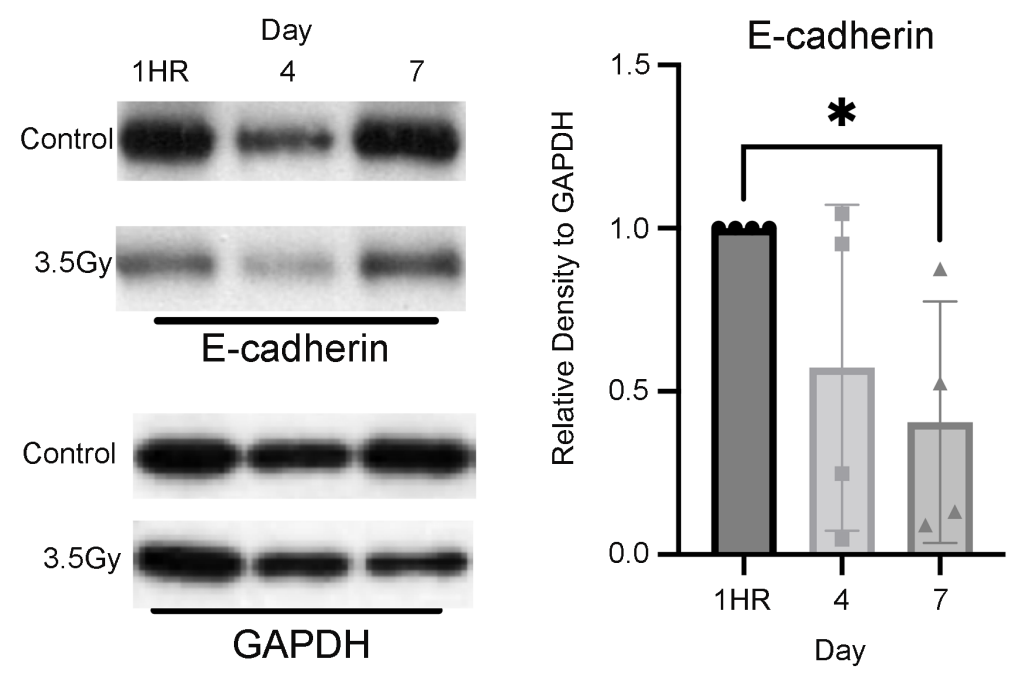

Supplemental Figure 5. Reduction of EMT-associated protein E-cadherin post-irradiation. Western blot of E-cadherin protein expression at 1 hour, day 4 and day 7 post-irradiation in control and 3.5Gy samples. GAPDH was used as the loading control. N = 3 and statistical analysis consisted of a one-way ANOVA with Tukey's correction for multiple comparisons.

Supplemental Table 1. Pro-fibrotic pathways and upstream regulators.

Pathway / Process / Regulator	Number of downstream gene targets	Z-score	$-\log_{10}$(BH) p-value	Number of downstream gene targets	Z-score	$-\log_{10}$(BH) p-value
	Day 2			Day 7		
Pulmonary Fibrosis Idiopathic Signaling Pathway	17	0.775	2.32319035	24	1.877	1.41237172
Extracellular matrix organization	11	0.302	4.96460698	9	1	1.16210297
Assembly of collagen fibrils	6	1.633	1.1197002	5	1.342	3.0962585
Hepatic Fibrosis	21	0	1.20125381	12	0	0.83619627
SMAD3	25	2.095	3.61730163	26	2.568	3.82421016
SMAD4	35	2.582	5.78144247	45	2.919	10.7612484
SNAI1	15	1.143	2.70839086	16	0.387	2.94322408
SNAI2	0	0	0	11	1.953	2.05403718
TGF beta	36	0.438	5.33313354	40	1.976	6.92916043
TGFβ1	113	2.078	8.69939088	144	3.479	19.4325154
TGFβ2	18	1.468	3.4465511	24	1.334	6.47395085
TGFβR1	10	1.177	1.9251388	11	1.16	2.14855399
TGFβR2	32	1.2	4.85062614	32	2.664	4.69676627
TWIST1	18	0.983	3.20171389	23	0.901	5.41851824
Fibrogenesis	43	-0.543	3.47269136	60	2.162	9.66812034
Fibrosis	98	0.143	8.22160462	104	0.596	9.58781214

Table containing the number of downstream target genes, z-score and $-\log_{10}$(BH) p-value for days 2 and 7, corresponding to **Figure 4A**.

Supplemental Table 2. Human primer sequences used for qPCR.

Gene	Forward	Reverse
ACTA2	CTATGCCTCTGGACGCACAACT	CAGATCCAGACGCATGATGGCA
ARPC2	CAGGATACAATGTCTCTTTGC	TCCCTATAATGGATAACTGCC
BAX	TGGAGCTGCAGAGGATGATTG	GAAGTTGCCGTCAGAAAACATG
CDKN1A	AGGTGGACCTGGAGACTCTCAG	TCCTCTTGGAGAAGATCAGCCG
COL1A1	GATTCCCTGGACCTAAAGGTGC	AGCCTCTCCATCTTTGCCAGCA
CTGF	CTTGCGAAGCTGACCTGGAAGA	CCGTCGGTACATACTCCACAGA
FN1	ACAACACCGAGGTGACTGAGAC	GGACACAACGATGCTTCCTGAG
GAPDH	GGAGCGAGATCCCTCCAAAAT	GGCTGTTGTCATACTTCTCATGG
MDM2	AGGCTGATCTTGAACTCCTAAAC	CAGGTGCCTCACATCTGTAATC
MiR-34a-5p	GGCAGTGTCTTAGCTGGTTGTAAAA	Universal (Norgen Cat# 54410)
NOTCH1	TGGACGACAACCAGAATGAG	CATCCAGGTGCTGCTGCGT
SNAI2	ATCTGCGGCAAGGCGTTTTCCA	GAGCCCTCAGATTTGACCTGTC
SNORD48	ATCTGCGGCAAGGCGTTTTCCA	Universal (Norgen Cat# 54410)
TWIST	GCCAGGTACATCGACTTCCTCT	TCCATCCTCCAGACCGAGAAGG
ZMAT3	GGGAAGAATCATGCCAAGAG	TTGACCCAGCTCTGAGGATT
18S	GCAGAATCCACGCCAGTACAAG	GCTTGTTGTCCAGACCATTGGC

Supplemental Table 3. Primary and secondary antibodies utilized for immunohistochemistry, Western blotting and immunofluorescence.

Antibody	Manufacturer	Catalogue #	Method Used	Dilution
E-cadherin	Cell Signaling Technology (Danvers, MA, USA)	3195	IHC	1:200
E-cadherin	Cell Signaling Technology (Danvers, MA, USA)	3195	WB	1:1000
Vimentin	Cell Signaling Technology (Danvers, MA, USA)	5741	IHC	1:200
Phospho H2AX	Cell Signaling Technology (Danvers, MA, USA)	9718	IF	1:500
MDM2	Invitrogen (Carlsbad, CA, USA)	PA5-13008	IF	1:25
MDM2	Cell Signaling Technology (Danvers, MA, USA)	51541	WB	1:1000
GAPDH	Cell Signaling Technology (Danvers, MA, USA)	2118	WB	1:1000
Goat anti-Rabbit IgG (H+L) Alexa Fluor 488	Invitrogen (Carlsbad, CA, USA)	A-11008	IF Secondary	1:500
Goat anti-Mouse IgG (H+L) Alexa Fluor 594	Invitrogen (Carlsbad, CA, USA)	A-11005	IF Secondary	1:500
