# Supplementary material for: A human ex vivo model of radiation-induced skin injury recapitulates p53-driven profibrotic response to radiotherapy
Source: JCI Insight. 2026 Mar 19;11(13):e198692. doi: 10.1172/jci.insight.198692 (PMC13460817; doi:10.1172/jci.insight.198692)
Supplement: Supplementary file 2 [file jciinsight-11-198692-s127.pdf]

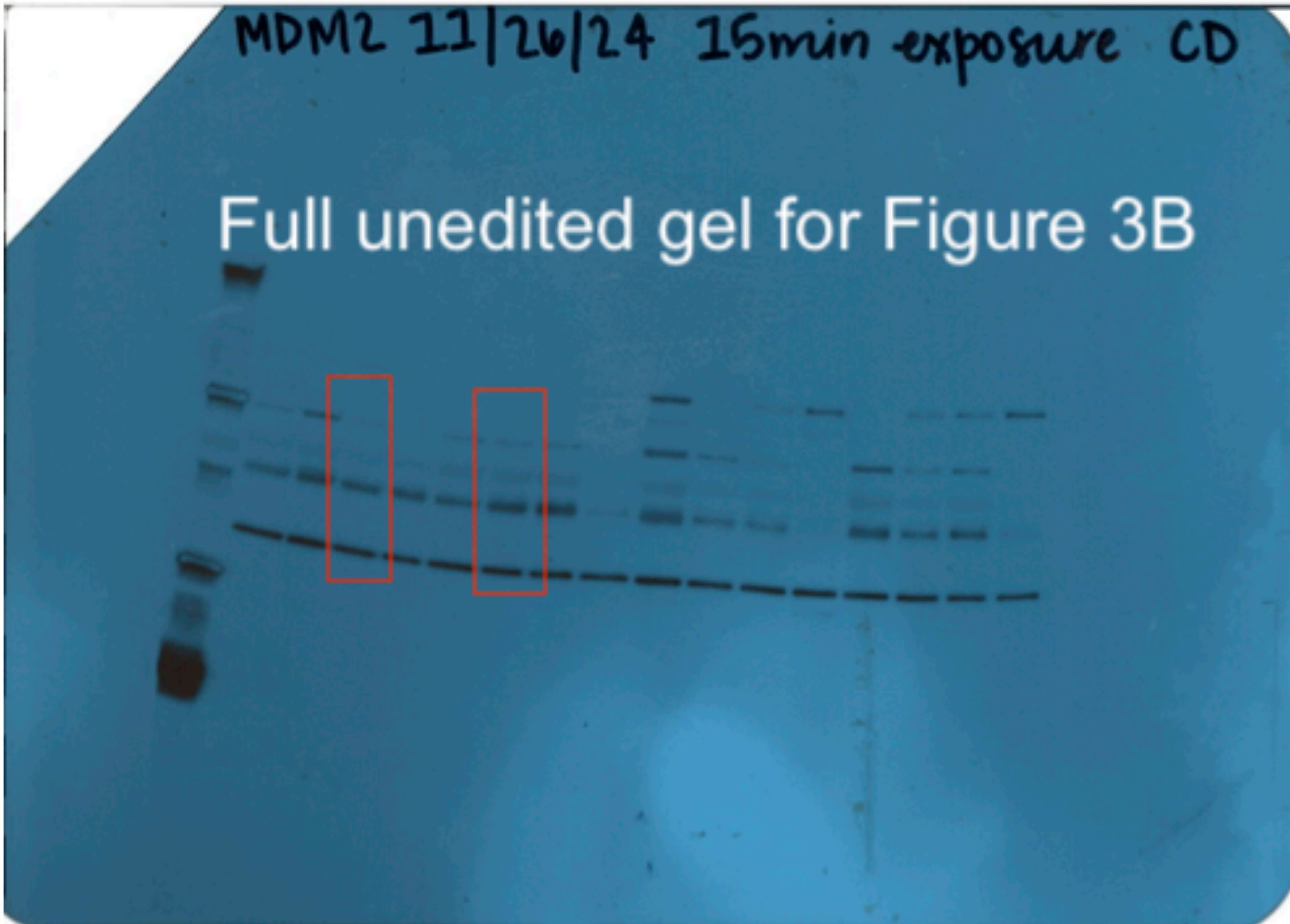
MDM2 11/26/24 15min exposure CD
Full unedited gel for Figure 3B

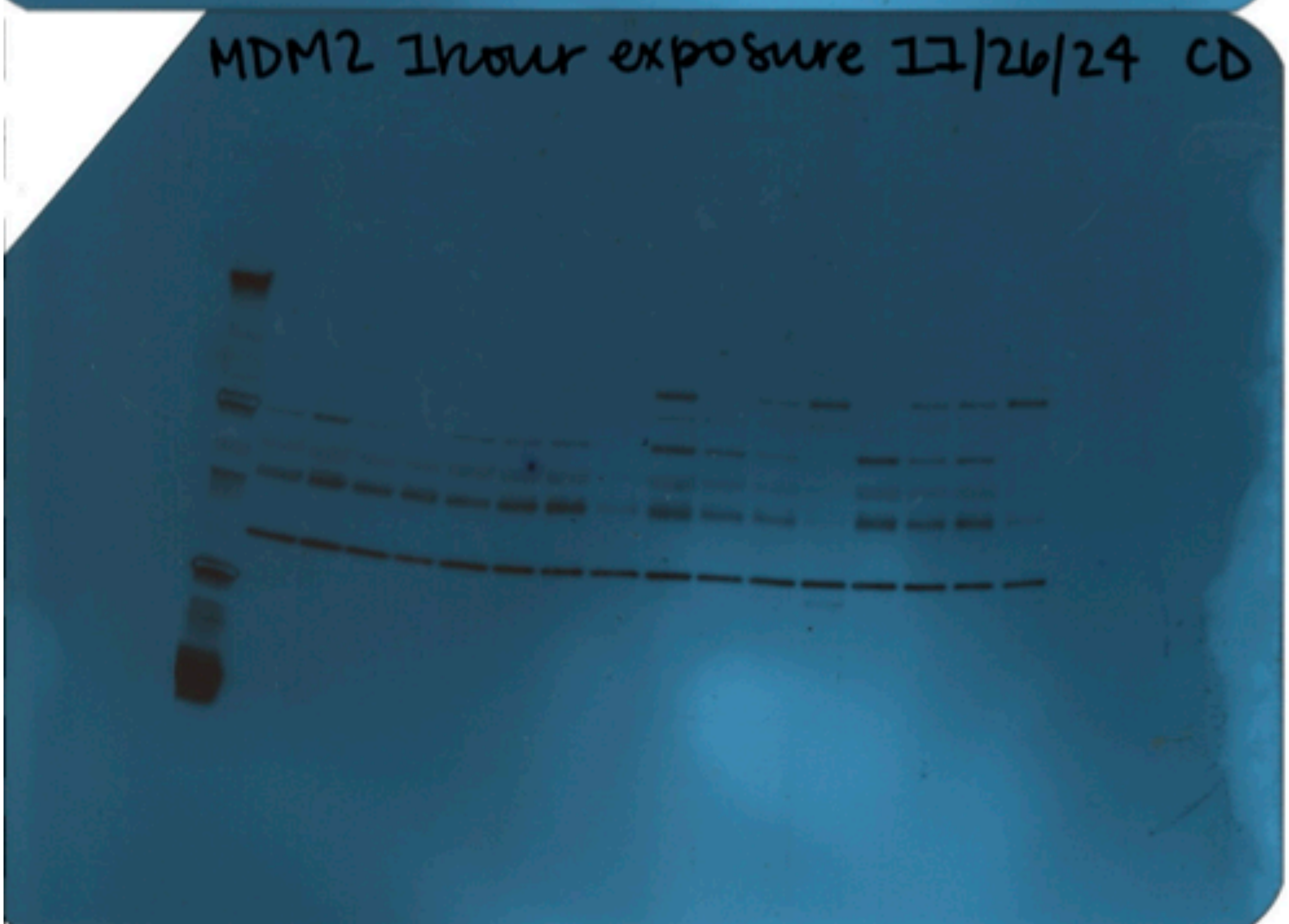
MDM2 1hour exposure 11/26/24 CD

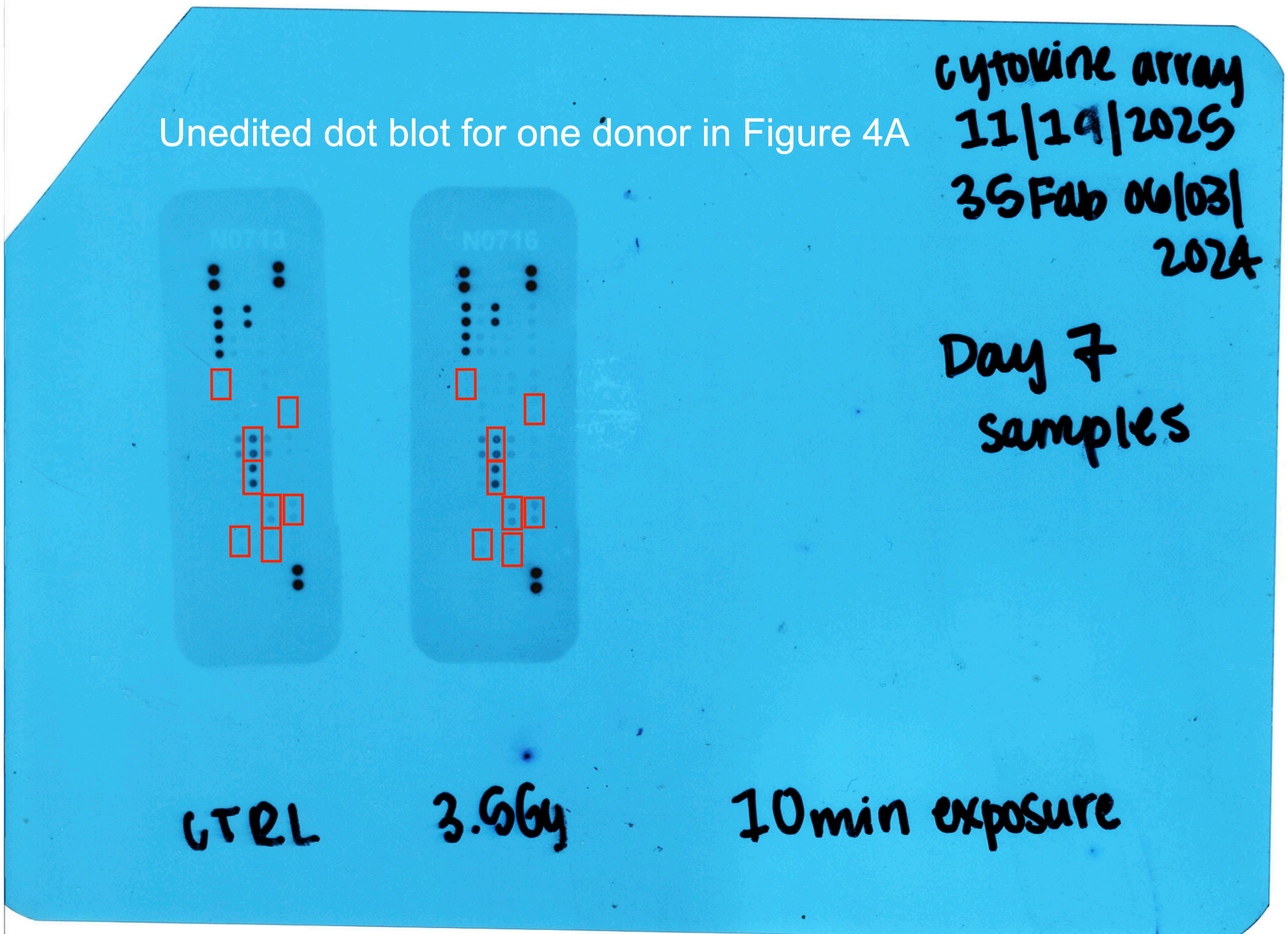

Unedited dot blot for one donor in Figure 4A

Unedited dot blot for one donor in Figure 4A

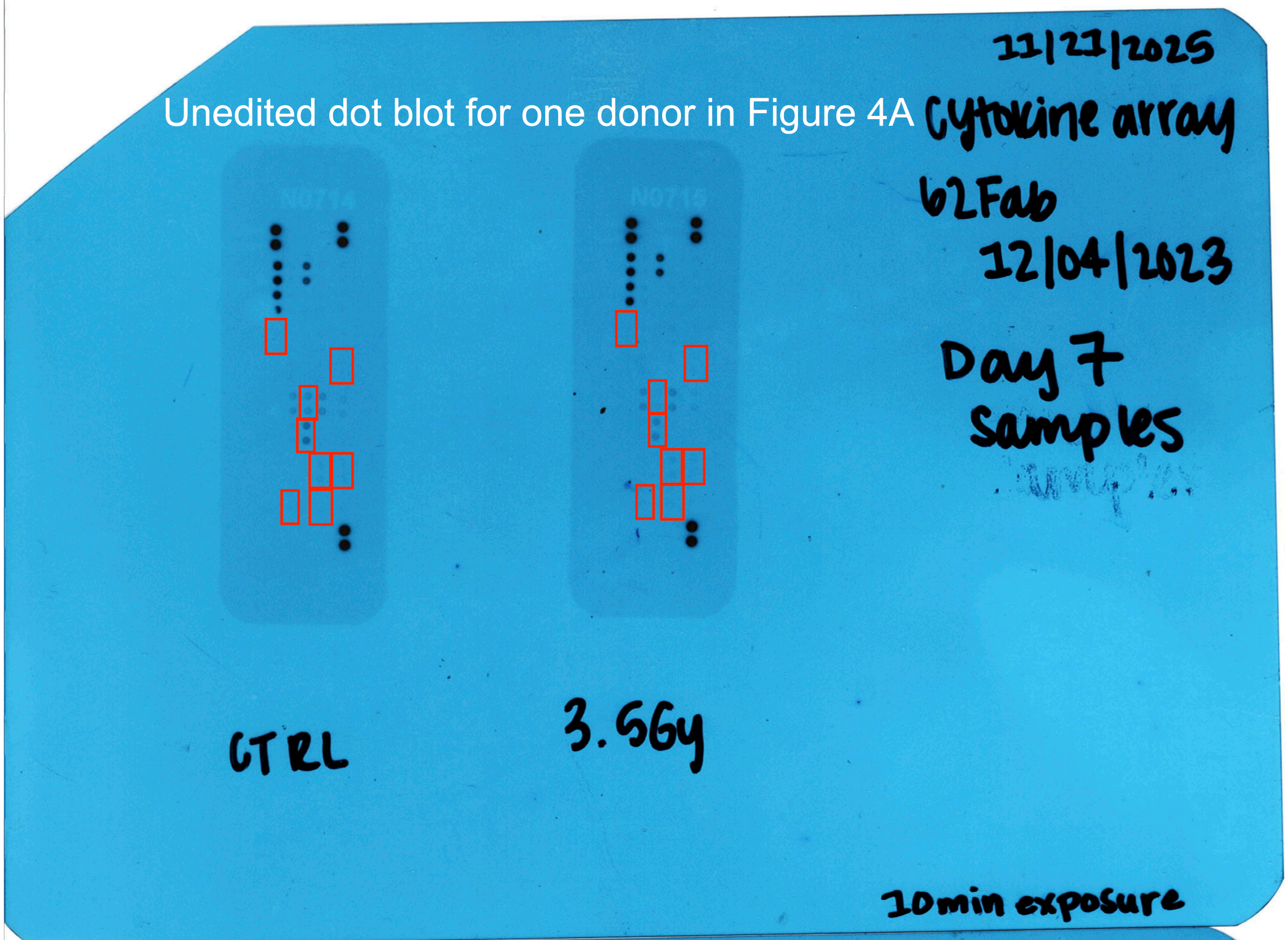

Unedited dot blot for one donor in Figure 4A

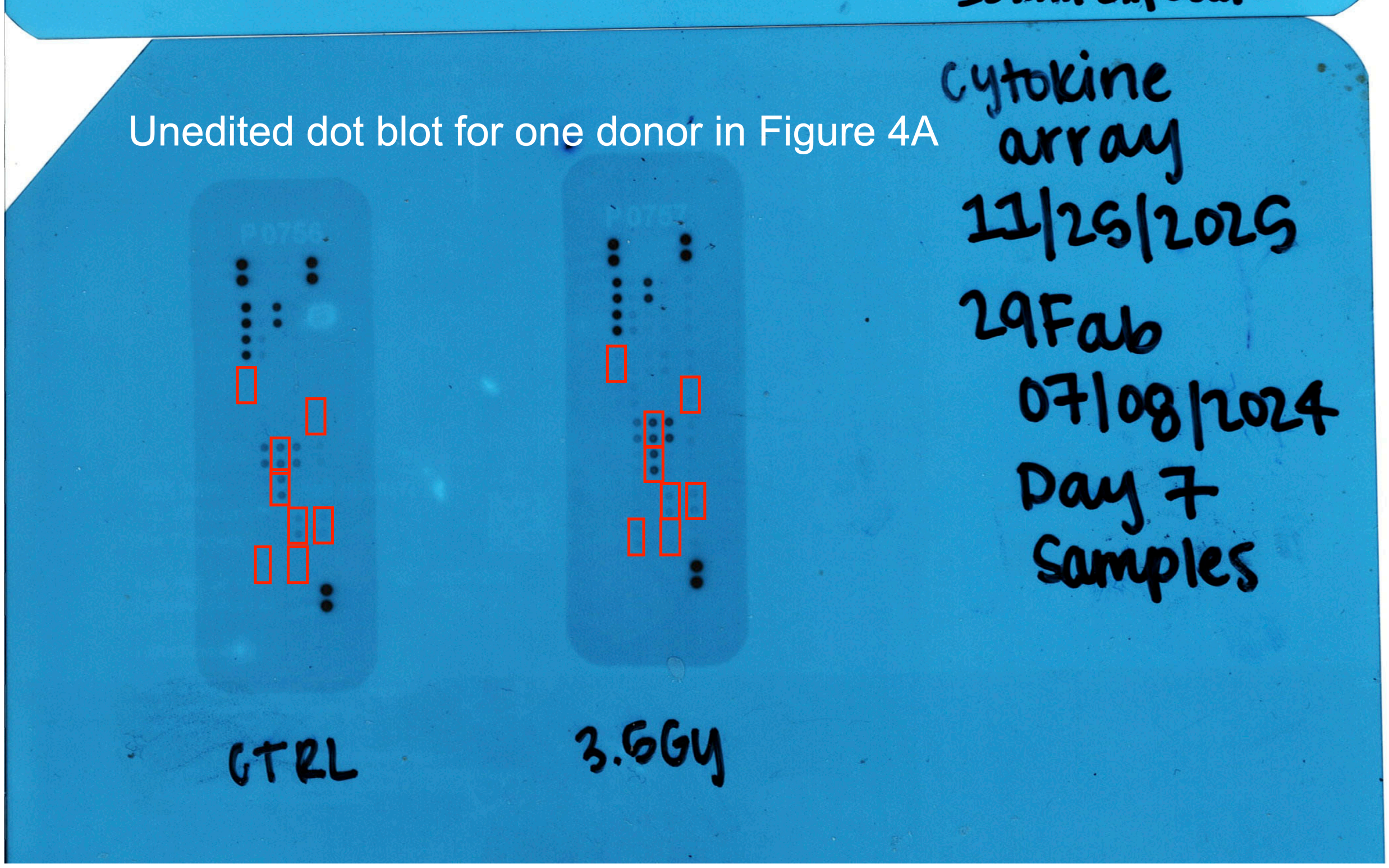

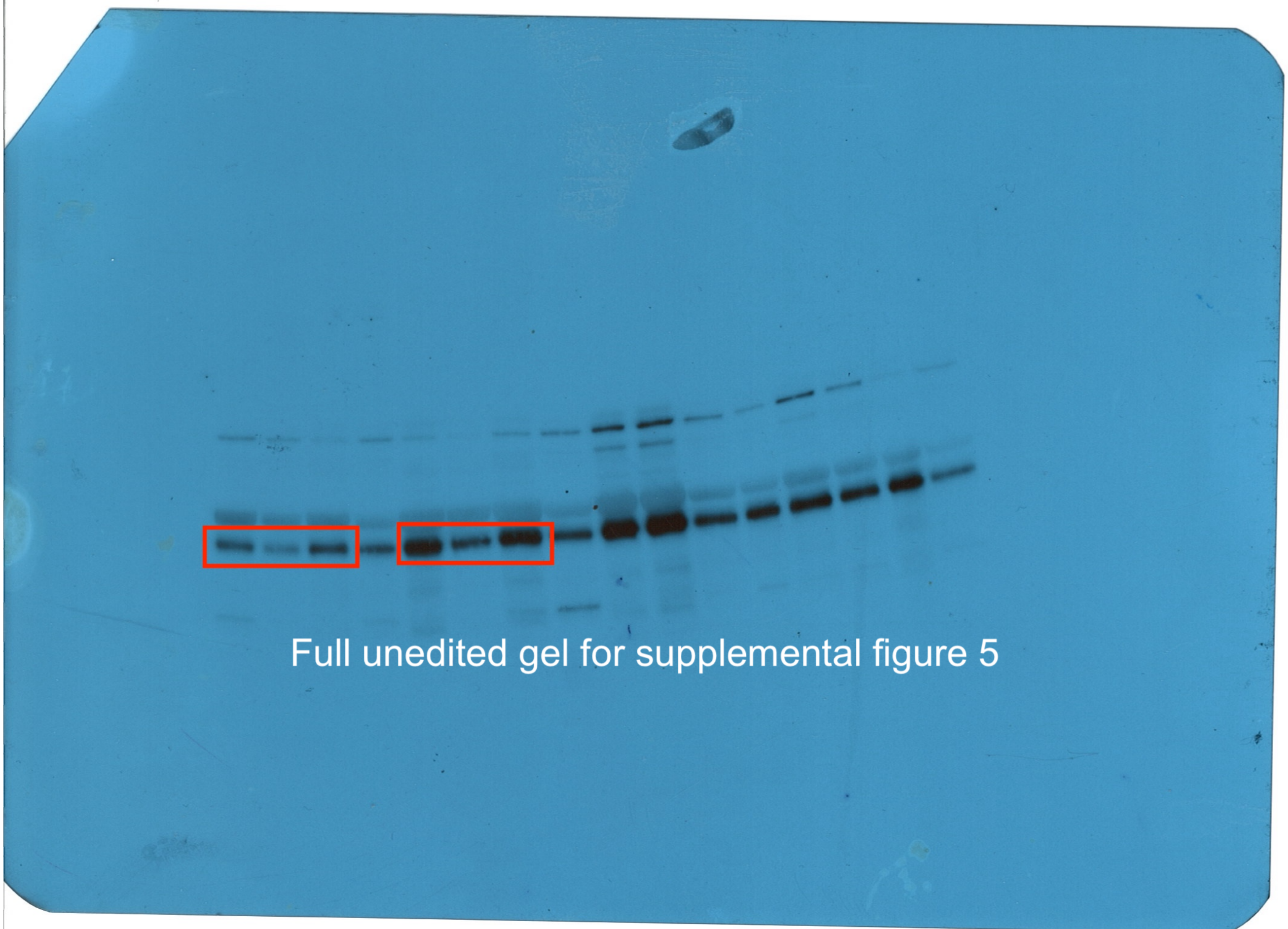

Full unedited gel for supplemental figure 5

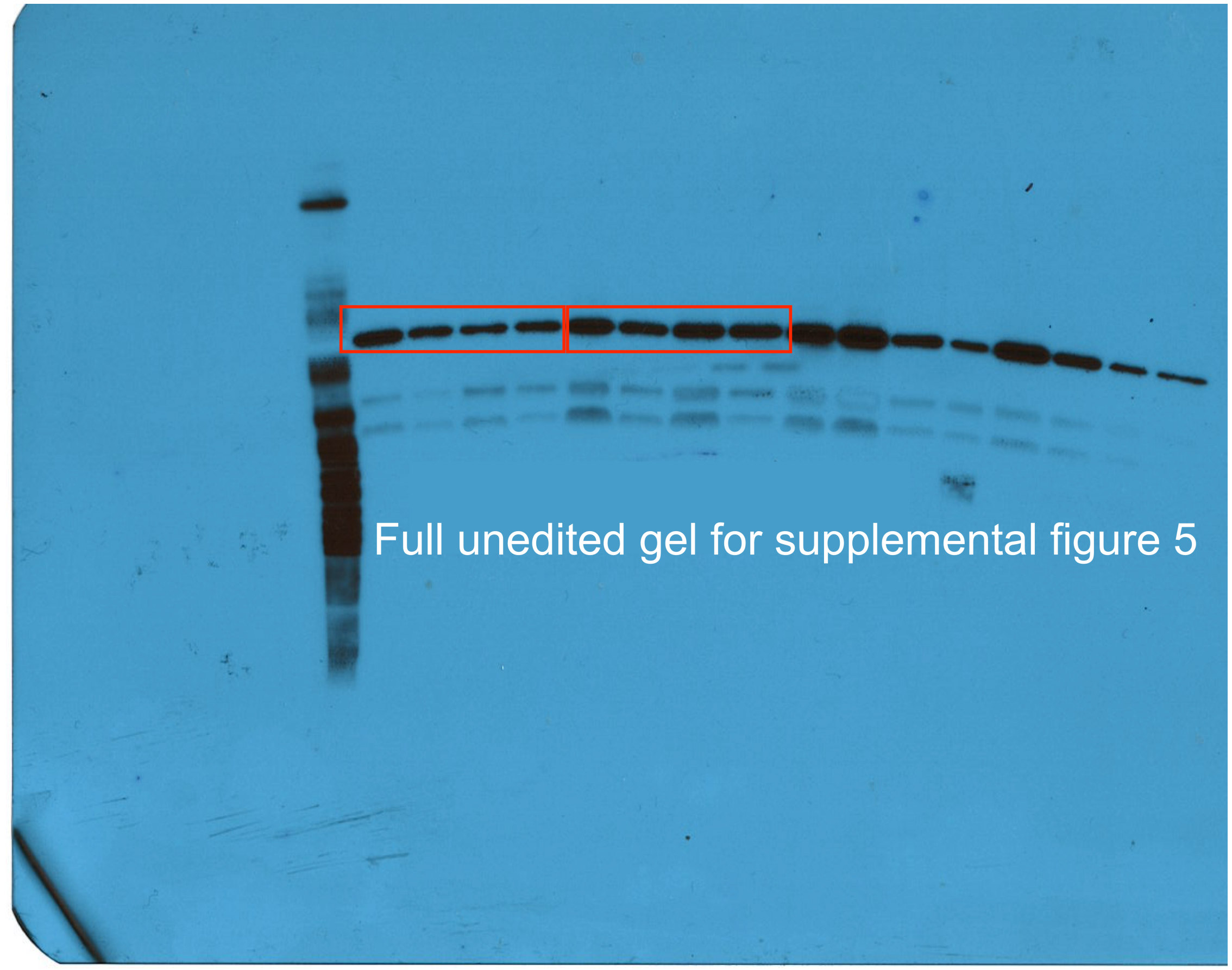
Full unedited gel for supplemental figure 5
